# Jahn‐Teller Effects in a Vanadate‐Stabilized Manganese‐Oxo Cubane Water Oxidation Catalyst

**DOI:** 10.1002/chem.202102539

**Published:** 2021-11-05

**Authors:** Sebastian Mai, Marcus Holzer, Anastasia Andreeva, Leticia González

**Affiliations:** ^1^ Institute of Theoretical Chemistry Faculty of Chemistry University of Vienna Währinger Straße 17 1090 Vienna Austria

**Keywords:** Density functional calculations, Jahn-Teller distortion, Polyoxometalates, Reaction mechanisms, Water splitting

## Abstract

Heuristic rules that allow identifying the preferred mixed‐valence isomers and Jahn‐Teller axis arrangements in the water oxidation catalyst [(Mn_4_O_4_)(V_4_O_13_)(OAc)_3_]^*n*−^ and its activated form [(Mn_4_O_4_)(V_4_O_13_)(OAc)_2_(H_2_O)(OH)]^*n*−^ are derived. These rules are based on computing all combinatorially possible mixed‐valence isomers and Jahn‐Teller axis arrangements of the Mn^III^ atoms, and associate energetic costs with some structural features, like crossings of multiple Jahn‐Teller axes, the location of these axes, or the involved ligands. It is found that the different oxidation states localize on different Mn centers, giving rise to clear Jahn‐Teller distortions, unlike in previous crystallographic findings where an apparent valence delocalization was found. The low barriers that connect different Jahn‐Teller axis arrangements suggest that the system quickly interconverts between them, leading to the observation of averaged bond lengths in the crystal structure. We conclude that the combination of cubane‐vanadate bonds that are chemically inert, cubane‐acetate/water bonds that can be activated through a Jahn‐Teller axis, and low activation barriers for intramolecular rearrangement of the Jahn‐Teller axes plays an essential role in the reactivity of this and probably related compounds.

## Graphical abstract

Jahn‐Teller effects in the manganese‐oxo vanadate catalyst [(Mn_4_O_4_)(V_4_O_13_)(OAc)_3_]^*n*−^ and its activated form [(Mn_4_O_4_)(V_4_O_13_)(OAc)_2_(H_2_)(OH)]^*n*−^ lead to a many local minima for different oxidation states and enhance the catalyst's reactivity.

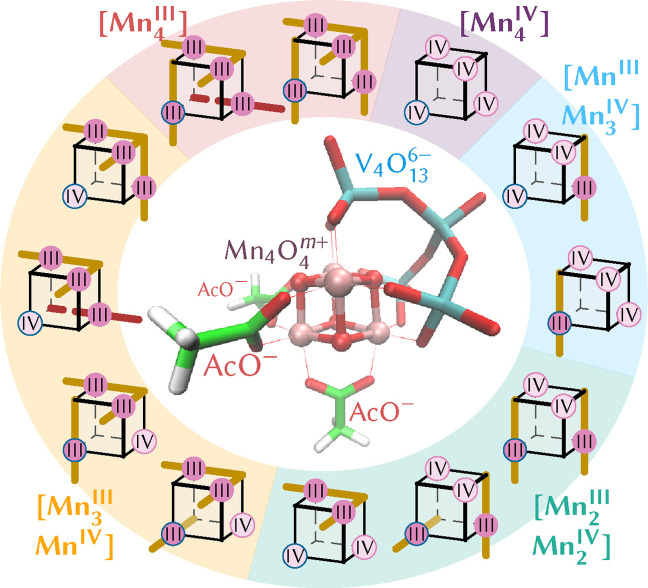

## Introduction

In photochemically driven water splitting[^1^, ^2^] (2 H_2_O$\overset{h\nu }{\to }$ O_2_+2 H_2_), several molecular components are required, including a photosensitizer, a hydrogen evolution catalyst, and a water oxidation catalyst (WOC). The need for good WOCs has motivated an immense research effort in the last decades,[^2^, ^3^, ^4^, ^5^] given the many requirements WOCs needs to fulfill. These include high catalytic activity, stability, and sustainability – like being free of rare metals, having oxidatively robust ligands, supporting multiple oxidation states, enabling proton‐coupled electron transfer (PCET), and others.^[2]^

One class of compounds able to catalyze the water oxidation reaction are manganese oxo clusters with a Mn_4_O_4_ cubane core.[^6^, ^7^, ^8^, ^9^, ^10^] They are related to the active site of natural photosystem II, which contains the oxygen evolving complex that features an Mn_4_CaO_5_ cluster. One very important aspect of the reactivity of this cluster is the availability of multiple oxidation states of the manganese atoms,^[11]^ where oxidation states +III and +IV are thought to play the central role.[^12^, ^13^, ^14^]

Simpler models to the oxygen evolving complex are Mn_4_O_4_ cubanes. The first such complex, with six diphenylphosphinate (Mn_4_O_4_L_6_, with L=O_2_PPh${}_{2}^{-}$ ) ligands, was reported by Dismukes and coworkers^[6]^ and further studied in the same group[^15^, ^16^, ^17^, ^18^] and by others.^[19]^ The structure of the compound was reported[^6^, ^19^] to show no discernible Jahn‐Teller (JT) distortions despite the formal presence of Mn^III^ atoms. In most cases, Mn^III^ in octahedral coordination has a high‐spin *d* ^4^ electron configuration, where one electron resides in the high‐energy $d_{z^{2}}$ or $d_{x^{2}-y^{2}}$ orbitals. This configuration is one of the text book examples of the JT effect,[^20^, ^21^, ^22^] where the coordination sphere is distorted to lower the energy of one of these orbitals. A complex containing Mn^III^ atoms without such JT distortions can be regarded as untypical^[22]^ (this is further discussed in Section “Comparison to X‐ray structure” below). The oxidized form of the complex by Dismukes and coworkers, Mn_4_O_4_L${}_{6}^{+}$ ,^[15]^ on the contrary, showed unequal bond lengths in the cubane crystal structure. It was also shown^[7]^ that simply increasing the electron‐donating abilities of the ligands can lead to the apparent localization of the Mn^III^ and Mn^IV^ oxidation states, introducing JT distortions to the cubane structure. Mn_4_O_4_ cubane structures with localized valences and JT distortions were also reported with six bidentate dimethylarsinic acid ligands,^[23]^ or in heteroleptic compounds with diphenylphosphinate, acetate, and a hexadentate pyridine/alcohol ligand.[^10^, ^24^, ^25^] JT distortions were also found in manganese‐oxo compounds like adamantane structures^[26]^ and others,^[27]^ as well as in Mn_4_O_3_Cl complexes^[28]^ or Mn_12_ structures,^[29]^ just to name a few. In passing we note that the JT effect is also intensely discussed in relation to the oxygen evolving catalyst, see, e. g., Refs. [12, 14, 30–33].

**Figure 1:**
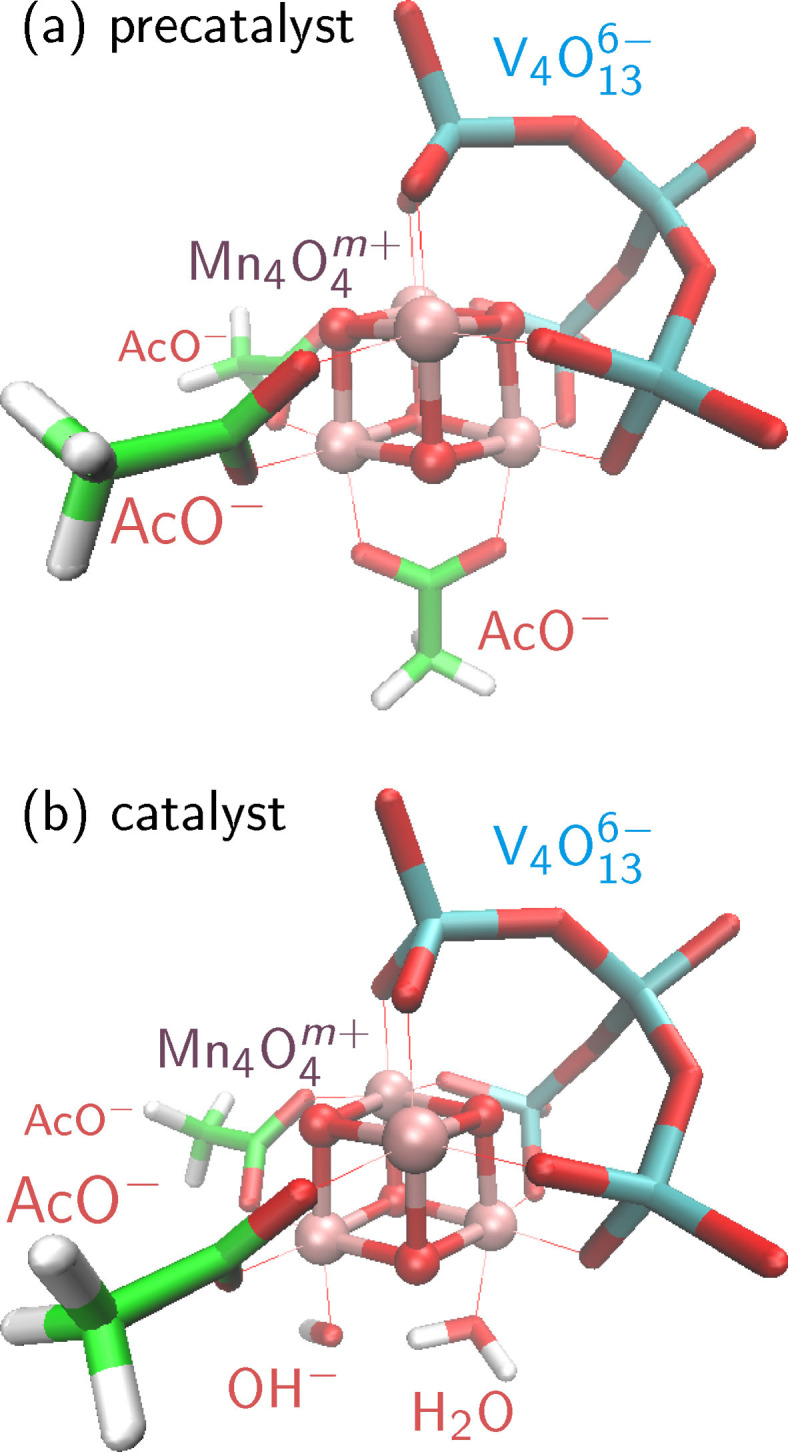
Depiction of the MnV WOC precatalyst with three acetate ligands (a) and the catalytically active form (b), where one of the acetates is exchanged for a water ligand and a hydroxide ligand. Mn atoms are colored pink, V teal, O red, C green, and H white.

**Figure 2:**
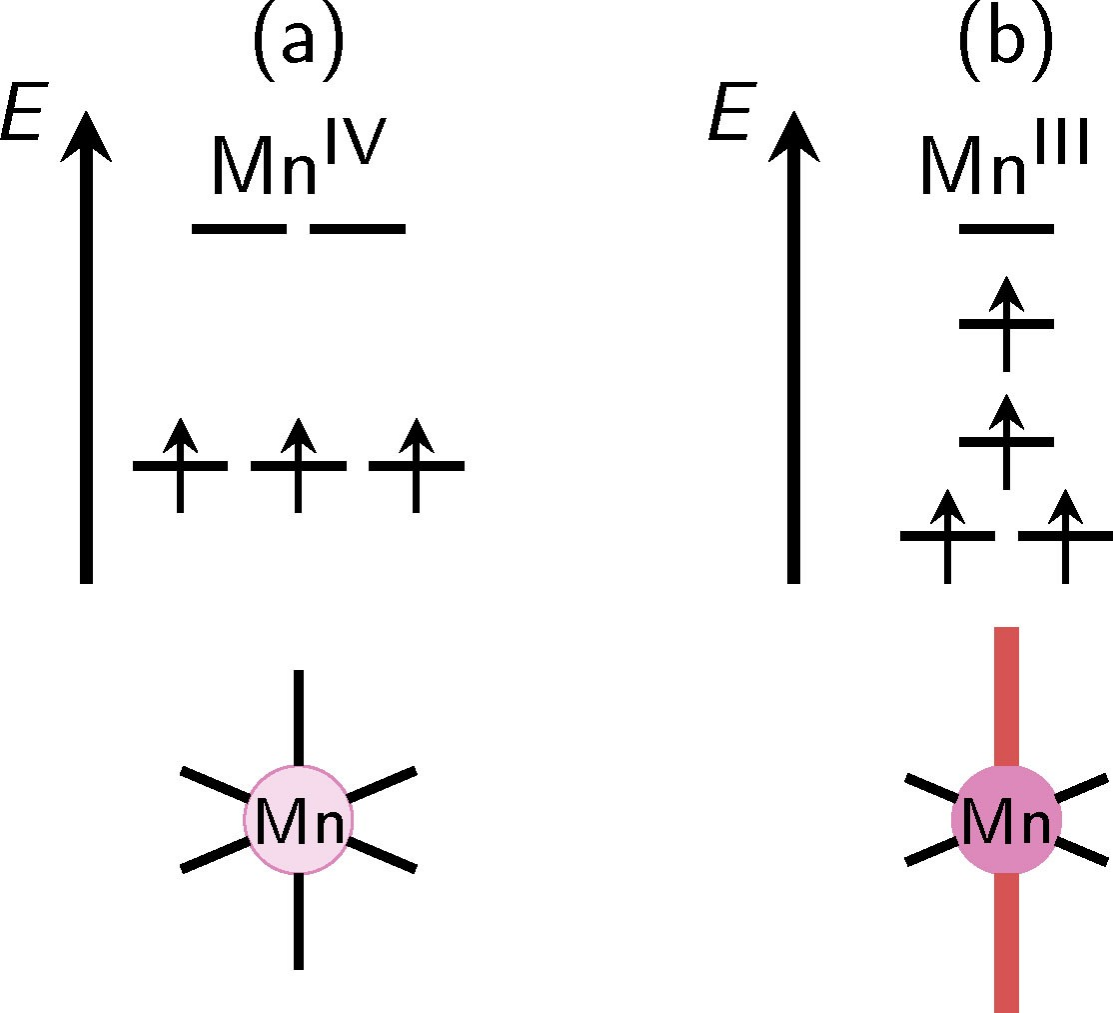
Orbital energy scheme for an ideal octahedrally coordinated Mn^IV^ center (a) and a Mn^III^ center with elongated octahedral coordination due to the JT effect that splits the two highest orbitals (b).

A recent catalyst based on a Mn_4_O_4_ cubane is the polyoxometalate‐stabilized compound^[9]^ [(Mn_4_O_4_)(V_4_O_13_)(OAc)_3_]^3−^, displayed in Figure 1a (and called “MnV WOC” in the following). This complex was able to effectively catalyze water oxidation in acetonitrile when coupled to a compatible photosensitizer, achieving a turn‐over frequency of 3.6 s^−1^ and a turn‐over number of approximately 12,000.^[34]^ Part of this success is probably due to the stabilizing effect of the tetravanadate ligand that is quite redox inactive. However, the activation mechanism and catalytic reaction cycle are still not well understood. Recent work^[35]^ has shown that the catalytically active structure (Figure 1b) is formed after two oxidation steps (producing a formal oxidation state of [Mn${}_{4}^{\mathrm{IV}}$ ]) and the exchange of one acetate ligand for a water molecule and a hydroxide ion. It was further observed that during this ligand exchange, an electron transfer between two Mn centers accompanied by a rearrangement of the JT axes contributed to significantly lowering the barrier for the rate‐determining step. One could therefore conclude that the localization of Mn^III^ and Mn^IV^ oxidation states as well as the orientation of JT axes of the Mn^III^ centers (see Figure 2) significantly influences the reactivity of the MnV WOC.

Here, we thus investigate the influence of mixed‐valence isomerism and the JT effect on the structural stability of the MnV WOC for all oxidation states between [Mn${}_{4}^{\mathrm{IV}}$ ] and [Mn${}_{4}^{\mathrm{III}}$ ]. Mixed‐valence isomerism (i. e., at which positions are the Mn^III^ atoms) and JT orientational isomerism (in which direction are the JT distortions pointing) have been discussed as two separate aspects in the literature.[^33^, ^36^] However, specifying the position of the JT axes automatically defines the position of the Mn^III^ atoms, and therefore below we discuss both aspects together as “JT axis arrangements”. We identify the preferred JT axis arrangements from all the possible combinations of location of the Mn^III^ atoms and all JT axis orientations within the point group symmetry of the MnV WOC. We also investigate the interactions between the different JT orientations and the effects of substituting one acetate ligand by a water molecule and a hydroxide ion, as is observed in the activated catalyst. Furthermore, we revisit the question whether the oxidation states in the MnV WOC are localized or delocalized, by comparing our findings to previously published^[9]^ X‐ray structures. The results are condensed into five heuristic rules that allow the general prediction of preferred JT axis arrangements in this and related complexes, both along the activation of the catalyst or its catalytic cycle. Further, it is expected that these rules inspire additional fundamental work on the role of JT effects in metal‐oxo cubanes.

## Methods

### Notation

We distinguish two species of the MnV WOC. The first is the unactivated precatalyst,^[9]^ which consists of the Mn_4_O_4_ cube, the V_4_O_13_ vanadate ligand, and three acetate ligands, which we will label “precatalyst” in the following. The second species is the activated catalyst,^[35]^ which is obtained from the precatalyst by release of one acetate ligand and attachment of one H_2_O ligand and one OH^−^ ligand. This species will be referred to as “catalyst” below. Both structures are shown in Figure 1. Note that the central proton of the catalyst can easily move between the H_2_O and OH^−^ ligands; thus, our investigations will include both tautomers.

Table 1 presents the investigated oxidation states. Note that for both precatalyst and catalyst the total charge of the ligands is identical, and therefore the table is valid for both species. The Kok‐analogous labels,^[11]^ where *S*_3_ corresponds to an [Mn${}_{4}^{\mathrm{IV}}$ ] oxidation state and the *S*_−1_ to an [Mn${}_{4}^{\mathrm{III}}$ ] oxidation state, are also included. The *S*_–1_ state was not part of the originally proposed Kok cycle,^[11]^ but such “super‐reduced” states are nowadays discussed for the oxygen evolving complex^[14]^ and might also be of relevance in the MnV WOC. The simpler labels, like “Mn4444”, clearly specify the oxidation states of the different Mn atoms and therefore will be used below.

**Table 1:** Considered oxidation states, corresponding labels, total charges, and total multiplicities.

Label	Label	*n*	*n*	Charge	Mult.
(Kok)		(Mn^IV^)	(Mn^III^)		2S +1
*S*_3_	Mn4444	4	0	−1	13
*S*_2_	Mn3444	3	1	−2	14
*S*_1_	Mn3344	2	2	−3	15
*S*_0_	Mn3334	1	3	−4	16
*S*_–1_	Mn3333	0	4	−5	17

**Figure 3:**
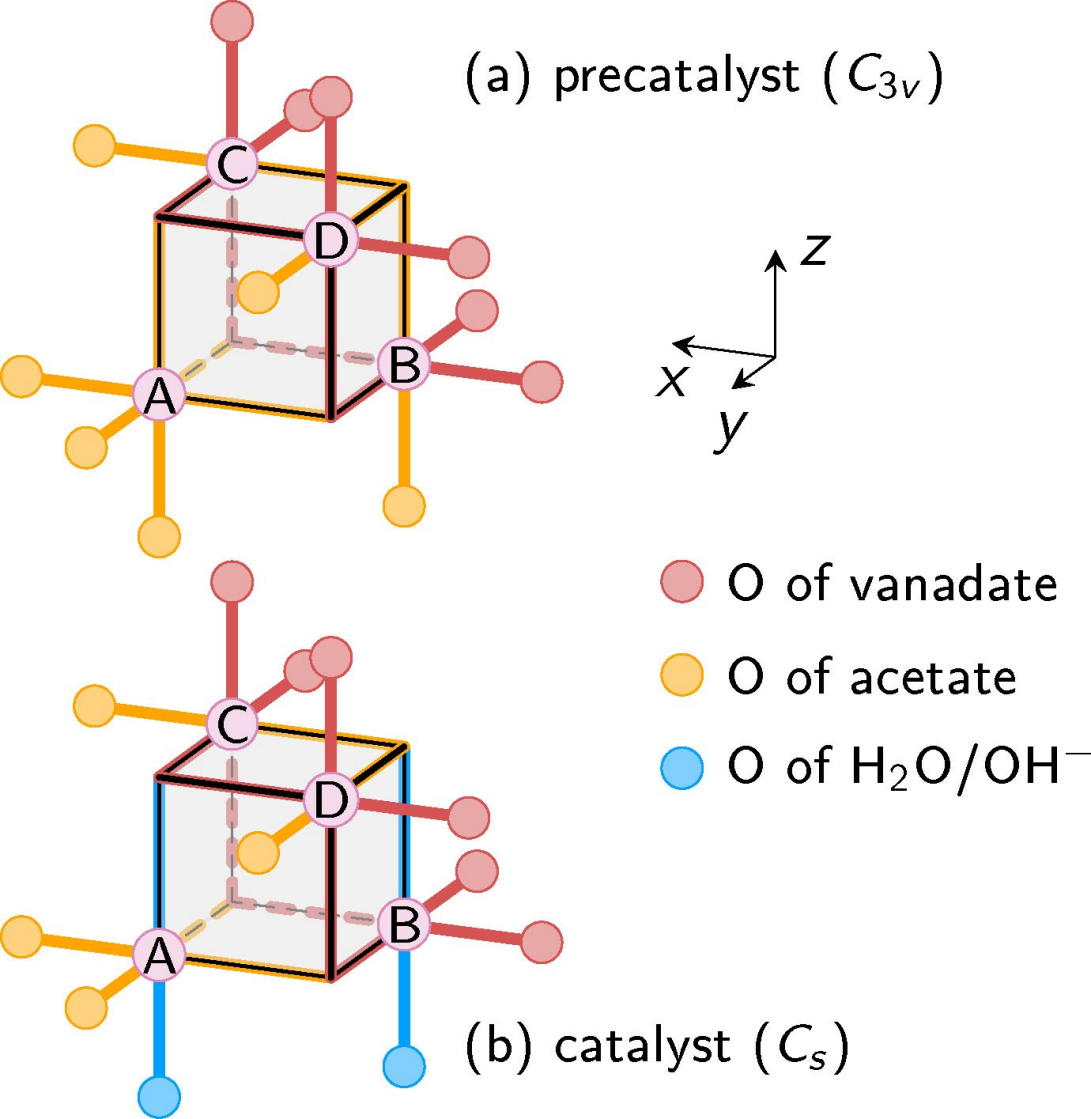
Schematic depictions of the central cube structure of the MnV catalyst and atom labels. (a) The precatalyst with three equivalent acetate ligands. (b) Activated catalyst with two acetate ligands and one pair of H_2_O/OH^−^ ligands. The orientation of the coordinate system is also depicted.

A thorough discussion of the JT distortions of the molecule requires that we define unique labels for each JT axis and all Mn atoms. Figure 3 shows the central cube structure of the precatalyst and activated catalyst. It also introduces the atom labels *A*, *B*, *C*, and *D*. Here, atom *A* is distinguished from the others by the fact that it is not bonded directly to the vanadate ligand. We refer to this atom *A* as the “apical” Mn atom (as will be discussed below, this atom behaves significantly different than the three other Mn atoms). The O atom opposite to *A* is analogously referred to as the apical O atom. The other Mn and O atoms are referred to as “non‐apical”.

As can be discerned in Figure 3, the precatalyst with three equal acetate ligands exhibits *C*_3*v*_ symmetry (in the absence of JT distortions). The rotation axis passes through the apical Mn and O atoms, whereas each of the three mirror planes passes through these two atoms and one of the Mn atoms *B*/*C*/*D*. On the contrary, the activated catalyst has approximate *C_s_* symmetry, with the only mirror plane passing through the apical Mn and O atoms, Mn atom *B*, and the O atoms of water and hydroxide. Note that in principle the positions of the H_2_O/OH^−^ protons can break this symmetry, but for the purpose of labeling the different JT distortions, we will ignore these protons. While we consider symmetry to identify all non‐equivalent JT distortions, we did not employ explicit symmetry in the computations.

With regard to the JT distortions, each of the four Mn atoms can be in exactly one of four different states, either being an Mn^IV^ atom without a JT axis, or being an Mn^III^ atom with a JT axis in *x*, *y*, or *z* direction. Hence, we build the labels for the different JT orientations from the characters “4”, “x”, “y”, and “z”. For example, the label “z444” indicates that atom *A* is a Mn^III^ with JT axis in *z* direction, and atoms *B*, *C*, and *D* are Mn atoms with oxidation state 4, i. e., Mn^IV^ atoms. Additionally, for the catalyst, we add an “O” or “H” prefix to the label, where “O” indicates that the OH^−^ ligand is attached to atom *A* and “H” indicates that H_2_O is attached to atom *A*. For example, the structure shown in Figure 1b would be labeled “O4444”.

### Optimization strategy

As we aim at finding how the different JT axes of the two molecules are arranged in all stable local minima, we generate starting geometries for all possible, symmetry‐nonequivalent JT distortions, which are then optimized. The starting geometries were produced from constrained optimizations, where the JT distortions were enforced by constraints. Mn−O bonds involved in the JT distortions were constrained to 2.10–2.30 Å, which is significantly longer than the Mn−O bonds of Mn^IV^ and helps to converge the initial wave function to the correct localization. These preoptimizations were carried out with loose optimization criteria and with smaller basis sets, as detailed below.

Subsequently, the preoptimized geometries were taken as initial geometries for unconstrained optimizations. The JT distortions of the resulting geometries were classified according to the involved 24 bond lengths (12 in the cube and 12 towards the ligands). For all distinct geometries, the optimization was followed by a frequency calculation to confirm the minimum and to compute the free energy of the minima including vibrational corrections. If the same geometry was reached in multiple optimizations from different preoptimized structures, then only one frequency calculation was performed.

### Electronic structure

The preoptimizations were carried out with the ORCA 4.2.1 package.[^37^, ^38^] We used the BP86 functional[^39^, ^40^] and the ZORA‐def2‐SVP[^41^, ^42^] basis set with the zeroth order regular approximation (ZORA).[^42^, ^43^, ^44^] The calculations also employed the D3 dispersion correction^[45]^ and the C‐PCM implicit solvation model^[46]^ to describe the acetontrile solvent used experimentally.^[9]^

The final optimizations and frequency calculations were performed with Gaussian 16,^[47]^ after we observed inconsistencies in the C‐PCM final energies obtained with ORCA. In Gaussian, we employed the BP86 functional, the def2‐SVP^[41]^ basis set for C and H, and the larger def2‐ZTVP^[41]^ basis set for Mn, V, and O. The GD3 dispersion correction^[48]^ was included. The effect of the acetonitrile solvent was described through the IEFPCM formalism.^[49]^

The functional was chosen based on several recommendations from literature[^13^, ^14^] that showed that the dispersion‐corrected BP86 functional can produce reasonable geometries for multi‐nuclear Mn complexes. We note that throughout the study, we have adhered to using unrestricted Kohn‐Sham calculations assuming a high‐spin configuration. Preliminary broken‐symmetry calculations indicated that the complex is likely to be in a low‐spin configuration, but that the energetic stabilization (about 1.3 kcal/mol for precatalyst Mn3344) from the broken‐symmetry calculation is virtually independent of the geometry. Hence, we assume that our findings (relative energies, barrier heights) are not affected by using the computationally more efficient high‐spin approach, as has been done previously on studies of the oxygen‐evolving complex.^[50]^

We note that several of the Gaussian 16 optimizations showed convergence problems close to the minimum. These were solved by trying various SCF and geometry optimization schemes, with the minimum confirmed after convergence by a frequency calculation.

We performed additional calculations to compute potential energy scans for transitions between different minima of the precatalyst in the Mn3344 oxidation state. For these calculations, transition states were obtained with the climbing‐image nudged elastic band method^[51]^ implemented in ORCA 4.2.1 and a subsequent transition state optimization. These optimizations were performed with the ORCA settings given above, except that for Mn, V, and O we used the ZORA‐def2‐TZVP basis set and employed the Gaussian point charge scheme^[52]^ for C‐PCM to eliminate the inconsistencies mentioned above. Subsequently, linear interpolation in Cartesian coordinate scans from minimum to transition state to minimum were performed, where for some scans the climbing image was used because the transition state optimization was not successful. To ensure comparability to the other reported energies, the scans were performed with Gaussian 16, using the settings given above.

### Generation of the symmetry‐nonequivalent starting geometries

In principle, the number of possible JT axis configurations can be computed in a combinatorial way. As there are four Mn atoms and each can be in any of four states (Mn^IV^ with no axis or Mn^III^ with either *x*, *y*, or *z* axis), there are in total $4^{4}=256$ possible JT arrangements for each molecule. This implies that, for the precatalyst and the catalyst (both tautomers), a total of 3×256=768 possible arrangements need to be considered. Molecular symmetry allows significantly reducing this large number of structures to be optimized. As the precatalyst exhibits *C*_3*v*_ symmetry, the JT arrangements are up to 6‐fold degenerate. The catalyst only exhibits *C_s_* symmetry, giving many arrangements that are two‐fold degenerate.

For example, for Mn3444, there is a threefold degenerate arrangement where the only JT axis is localized on the apical Mn atom (labeled z444, y444, and x444), a threefold degenerate arrangement where the JT axis is on *B*/*C*/*D* and points towards an acetate (4z44, 44x4, 444y), and a sixfold degenerate arrangement with the JT axis on *B*/*C*/*D* pointing towards the vanadate (4x44, 4y44, 44y4, 44z4, 444x, 444z). These 12 possibilities are shown in Figure S1 in the Supporting Information. The entire list of all symmetry equivalences is given in Tables S1 and S2 in the Supporting Information.

For the precatalyst, considering symmetry there is a total of 52 symmetry‐nonequivalent JT arrangements. This includes 1 arrangement for Mn4444, 3 arrangements for Mn3444, 11 for Mn3344, 22 for Mn3334, and 15 for Mn3333. These 52 arrangements are presented in Figure S2. For each of the two tautomers of the catalyst, there are 136 symmetry‐nonequivalent JT arrangements (1 for Mn4444, 7 for Mn3444, 29 for Mn3344, 57 for Mn3334, 42 for Mn3333). In order to make this still large number more manageable, based on the results from the precatalyst (see below), we decided to omit the optimization of all JT arrangements of Mn3334 and Mn3333 with JT axes pointing towards the vanadate ligand. This reduces the number of JT arrangements for Mn3334 to only 6 and for Mn3333 to only 2, giving a total of 45 investigated JT arrangements for each tautomer. These JT arrangements for all oxidation states of the catalyst are shown in Figures S3 (OH^−^ on apical position) and S4 (H_2_O on apical position).

In total, we considered 142 starting geometries including both molecules, all tautomers, and all oxidation states.

## Results and discussion

### Jahn‐Teller axis arrangements

During the pre‐optimizations and full optimizations, we monitored the convergence to the different minima through the averages of the bond lengths of all the 12 coordination axes of the four manganese atoms. The full set of average bond lengths after pre‐optimization and after full optimization is given in Tables S3, S4, and S5 in the Supporting Information. Using these average bond lengths, one can automatically identify non‐JT and JT axes in the optimized structures. Non‐JT axes are consistently in the 1.88 Å–1.97 Å range, whereas JT axes showed an average bond length of 2.17–2.32 Å.

**Figure 4:**
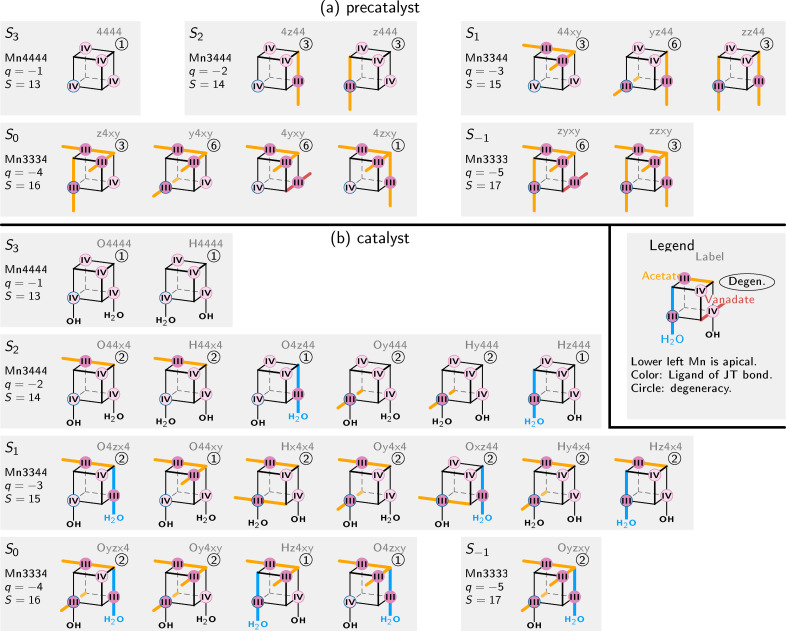
Overview over all JT axis arrangements of the precatalyst (a) and the catalyst (b) that are stable minima. Each block corresponds to one overall oxidation state (*S*_3_, *S*_2_, . . . ; indicated by charge *q* and spin *S*). Each cube is oriented in the same way, with the apical Mn atom in the lower‐left corner. JT axes are indicated by colored, thick lines, with the color indicating the type of bonded ligand. The degeneracy of the arrangement and a label are also given. For reference, at the bottom we also show the four basic relative orientations between two JT axes.

The 142 optimizations lead to 32 different local minima, collected in Figure 4 (see also Figures S2, S3, and S4). The Cartesian coordinates of all 32 minima are collected in two supplementary files in XYZ format. For the precatalyst, there are 12 stable minima (Figure 4a). For Mn4444, the optimization lead to a fully *C*_3*v*_ symmetric geometry. For Mn3444, we found two different minima, one where the JT axis is located on a non‐apical Mn (4z44) and one where the JT axis is located on the apical Mn atom (z444). All attempts to optimize a structure with the JT axis pointing towards the vanadate ligand failed, indicating that such JT axes are energetically unfavorable and lead barrierlessly to minima with the JT axis towards an acetate. Likewise, for Mn3344 we did not observe a stable minimum with a vanadate JT axis. Three different minima with two acetate JT axes are obtained, one where the JT axes intersect at the apical O atom (“crossing” arrangement), and two where one JT axis is on the apical Mn atom (a “skew” and a “parallel” arrangement). These motifs – intersecting JT axes and a JT axis on the apical Mn – are also found in the Mn3334 and Mn3333 oxidation states. However, here we also find few stable minimum with one vanadate JT axis.

For the catalyst, the findings are slightly more diverse due to the larger number of different ligands. For Mn4444, we obtained two stable minima that only differ in the position of the water proton. For Mn3444, a total of six structures was found, where all exhibit JT axes towards either an acetate or the H_2_O ligand. No structures with the JT axis to the vanadate or the OH^−^ were found to be stable. For Mn3344, seven different structures were found, out of the nine possible structures with JT axes to acetate or H_2_O; again, JT axes to vanadate or OH^−^ were not stable. For Mn3334 and Mn3333 of the catalyst, we only used starting structures without vanadate JT axes and found only four plus one minima.

Generally speaking, these findings strongly indicate that there are certain JT axis configurations that are energetically unfavorable, like JT axis pointing to the vanadate oxygens or towards the OH^−^ ligand.

### Relative energies in the precatalyst

The relative energies, degeneracies, and Boltzmann populations of the 12 stable minima for the precatalyst are given in Table 2. As can be seen, the energies of the different minima and the corresponding Boltzmann distributions differ substantially. For the precatalyst, in all oxidation states except Mn3334 there is only one minimum that is thermally populated at room temperature.

**Table 2:** Free energy differences between the different stable minima of the five oxidation states of the precatalyst.

Label	Conf.	Δ*G*	Degen‐	Population
		(kcal/mol)	eracy	(300 K)
Mn4444	4444	0.0	1	100 %
Mn3444	4z44	0.0	3	100 %
	z444	9.7	3	0 %
Mn3344	44xy	0.0	3	99.9 %
	yz44	3.9	6	0.1 %
	zz44	4.4	3	0 %
Mn3334	z4xy	0.0	3	35 %
	y4xy	0.05	6	64 %
	4yxy	2.2	6	1 %
	4zxy	3.2	1	0 %
Mn3333	zyxy	0.0	6	100 %
	zzxy	5.1	3	0 %

More interestingly, based on the results in Tables 2 and 3, we can find several heuristic rules that can be used to understand the different energies of the minima in a general fashion. The first rule can be obtained by comparing the energies of the two minima for the precatalyst in the Mn3444 oxidation state (*S*_2_). Here, the minimum with the JT axis at the apical Mn atom (z444) is almost 10 kcal/mol less stable than the minimum with the JT axis at the non‐apical Mn atom (4z44). This very large difference is not due to the ligand located along the JT axis, as in both cases this is an acetate O atom. Instead, it can be assumed that the ligands orthogonal to the JT axis are responsible for the difference in energy. The JT axis at the non‐apical atom is neighbored by two vanadate O atoms, whereas the JT axis on the apical atom is flanked by two acetate O atoms. As can be seen in Figure S5 for the 4444 state, the apical Mn atom exhibits a larger ligand field splitting (and higher unoccupied *d* orbitals), compared to a non‐apical Mn atom. Thus, JT distortions produce an energetically more favorable $d_{z^{2}}$ orbital at the non‐apical Mn atoms (in 4z44) and a less favorable one at the apical Mn atom (in z444).

**Table 3:** Free energy differences between the different stable minima of the five oxidation states of the catalyst.

Label	Conf.	Δ*G*	Degen‐	Population
		(kcal/mol)	eracy	(300 K)
Mn4444	O4444	0.00	1	61.2 %
	H4444	0.24	1	38.8 %
Mn3444	O44x4	0.00	2	59.3 %
	H44x4	0.37	2	29.4 %
	O4z44	0.51	1	11.3 %
	Oy444	7.46	2	0 %
	Hy444	7.67	2	0 %
	Hz444	7.70	1	0 %
Mn3344	O4zx4	0.00	2	73.0 %
	O44xy	0.22	1	24.0 %
	Hx4x4	2.00	2	1.7 %
	Oy4x4	2.63	2	0.5 %
	Oxz44	2.67	2	0.5 %
	Hy4x4	2.81	2	0.4 %
	Hz4x4	4.21	2	0 %
Mn3334	Oyzx4	0.00	2	94.0 %
	Oy4xy	1.52	2	5.3 %
	Hz4xy	2.22	1	0.7 %
	O4zxy	4.20	1	0 %
Mn3333	Oyzxy	0.0	2	100 %

For the second rule, we direct our attention to the three minima for the precatalyst in the Mn3344 oxidation state (*S*_1_). The lowest‐energy minimum (44xy) features two non‐apical JT axes that point towards acetate O atoms and cross at the apical O atom. The two other minima, about 4 kcal/mol above the lowest‐energy minimum, both feature an energetically unfavorable apical JT axis (yz44 and zz44). If we assume that the energetic cost of an apical JT axis is about 10 kcal/mol, then the 4 kcal/mol energy difference could be explained by a second rule that the crossing of two JT axes entails an extra energetic cost of approximately 6 kcal/mol. One possible explanation for this behaviour might be the unfavorable electrostatic repulsion due to a high electron density at the apical O atom from multiple occupied $d_{z^{2}}$ orbitals pointing in its direction. The high negative charge on the apical O atom at low oxidation states can be seen in Table S6. As the energies of the yz44 and zz44 minima are roughly identical, we can further conclude that a skew or parallel arrangement of two JT axes does not notably affect the energy (although parallel JT axes seem to be slightly less favorable).

We can now apply these two rules to the four optimized minima of the Mn3334 oxidation state (*S*_0_). The z4xy and y4xy minima both feature an apical JT axis as well as a two‐axes crossing, and differ only in the relative direction of the JT axes. Hence, they should have similar energies, in good agreement with the 0.05 kcal/mol difference obtained in the calculations. The two other minima (4yxy and 4zxy) do not have an unfavorable apical JT axis but nevertheless a higher energy. Hence, the presence of a JT axis towards a vanadate O (in 4yxy) and a three‐axes crossing (in 4zxy) appear to entail an additional cost. We can thus establish rule three, that a vanadate JT axis costs about 12 kcal/mol. Likewise, in rule four we state that a three‐axes crossing costs 13 kcal/mol more than a two‐axes crossing – 19 kcal/mol in total, which is almost exactly three times as much as a two‐axes crossing. The energetic cost of a vanadate JT axis seems to arise because the involved Mn−O_vanadate_ bonds appear to be stronger and more covalent than the other Mn−O bonds. Based on the orbital shapes shown in Figure S5 and the population analysis in Tables S6 and S7, the Mn−O_vanadate_ bonds involve a favorable oxygen *p* to metal *d* interaction that has a net bonding effect as the Mn *d* orbitals are less than half filled. The strength of these Mn−O bonds is also indicated by their short bond distance (see Tables S3 to S5 and the comparison to the X‐ray structure below). This favorable bonding interaction would be quenched by JT bond elongation, explaining why JT axes towards the vanadate are avoided.

For the Mn3333 state (*S*_−1_), in principle these four rules should be sufficient to predict the energetics, but the predicted energy difference (1 kcal/mol difference, as zyxy has a vanadate JT axis and zzxy has a three‐axes crossing) does not agree fully with the computed 5 kcal/mol energy difference. However, inspection of the optimized geometries shows that zyxy actually dissociates one acetate O atom, leading to a five‐coordinated Mn atom. It is worth noting that previous work[^53^, ^54^] has already discussed the presence and the effect of five‐coordinated Mn^III^ atoms in the oxygen evolving complex of photosystem II, in terms of a particularly strong JT effect.^[54]^ As it is difficult to estimate the possible effect of filling this vacancy with another solvent molecule (or keeping a five‐coordinated Mn atom), we prefer to not consider this minimum further.

With the four proposed rules, we can now estimate the energetic cost of those structures that turned out to not be stable minima. A table with all symmetry‐nonequivalent JT arrangements, their energy predicted by these rules, and the computed energies for comparison are given in Table S8 in the Supporting Information. For example, for Mn3444 there is one JT arrangement that did not lead to a stable minimum, containing one JT axis towards a vanadate. Hence, according to rule three, this hypothetical structure would have an energy of about 12 kcal/mol, higher than both of the stable minima for this oxidation state (0 kcal/mol for 4z44, 10 kcal/mol for z444). For Mn3344, out of the 11 possible JT arrangements only 3 were found to be stable, with predicted energies of about 6 kcal/mol (44xy) and 10 kcal/mol (yz44 and zz44). The four rules successfully predict that the 8 other hypothetical JT arrangements (see Figure S2) are all higher in energy (the structures 44xx and 44xz with predicted energies of about 12 kcal/mol would be the next lowest). The rules also work similarly for Mn3334, where other JT arrangements would be at least 3 kcal/mol higher in energy than any of the four stable minima.

These rules explain why vanadate JT axes – although energetically unfavorable – can appear for the low oxidation states: Those states are so overcrowded with JT axes that a high energetic cost comes from axes crossings or an apical JT axis. Hence, it might be energetically more favorable to accept a vanadate JT axis in order to get rid of three‐axes crossings or an apical JT axis, both of which are also energetically unfavorable. On the contrary, for the high oxidation states, JT axis arrangements without vanadate axes are energetically clearly favored.

### Relative energies in the catalyst

The analogous results on the relative energies of the catalyst are compiled in Table 3. As can be seen, in the activated complex, in most oxidation states the calculations predict a thermal mixture of distinguishable minima. In principle, the data in the table is consistent with the discussion above for the precatalyst. However, in order to explain all energies, we need to adjust some of the energetic costs and add at least one more rule.

The free energies given in Table 3 for the Mn4444 (*S*_3_) state show that in the absence of any JT axes, the position of the proton (O4444 versus H4444, see also Figure 4b) does not significantly affect the energy. The small energy difference of 0.2 kcal/mol indicates that probably no specific rule is necessary here. However, we note that all lowest‐energy minima for all oxidation states have the hydroxide ligand at the apical Mn atom and the water ligand at the non‐apical one.

For Mn3444 (*S*_2_), we observe again a very small energetic difference due to the position of the proton in two cases (O44x4 versus H44x4, Oy444 versus Hy444). We also see that the first rule found above – that an apical JT axis has a significant energetic cost – is nicely reproduced here. However, assessing the energies of the affected arrangements (Oy444, Hy444, Hz444) shows that for the catalyst the cost of the apical JT axis is slightly lower at about 7.5 kcal/mol (compared to 10 kcal/mol in the precatalyst). This is probably due to the different ligand field of the water/hydroxide ligands, compared to a third acetate ligand. For the same reason, we observe a differentiation between the non‐apical, non‐vanadate JT axes. As can be seen from comparing O44x4 and O4z44, an acetate JT axis and a water JT axis are slightly different (0.5 kcal/mol) in energy, with the acatete JT axis being slightly more favorable. Additional information can be gained by considering which JT arrangements are absent from the list of stable minima. As shown in Table S9 in the Supporting Information, based on the already established rules, there should be two more stable JT arrangements – H4z44 and Oz444. However, multiple attempts to optimize these minima failed and always lead to proton transfer (to the O4z44 and Hz444 minima). This strongly suggests that JT axes towards the hydroxide ligand entail an additional energetic cost, similar to the JT axes towards a vanadate ligand. However, our results do not allow quantifying the energetic cost of a hydroxide JT axis, as none of the optimized minima contained one. Hence, we can only conjecture a fifth rule, saying that hydroxide JT axes are energetically unfavorable. This rule could be explained by the fact that the ligand involved in a JT axis obtains a rather high electron density through the filled $d_{z^{2}}$ orbital, and this higher electron density makes the ligand more basic such that it is energetically more favorable to move the proton to the JT axis. We note that such movement of a proton towards the JT axis has been observed before in density functional theory (DFT) calculations.^[55]^

**Figure 5:**
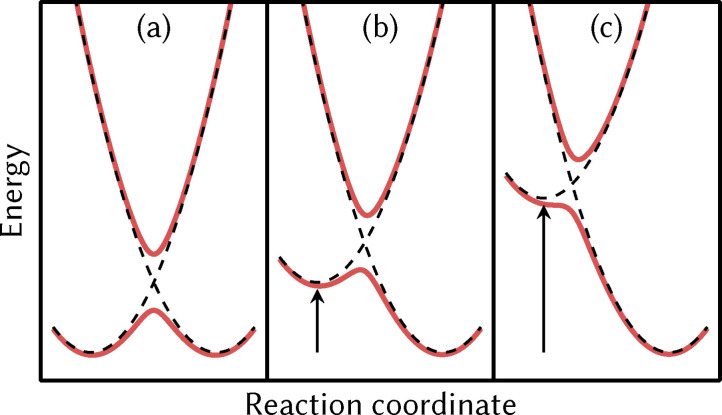
Simple two‐state diabatic model explaining how energy shifts of a minimum can reduce and remove a transition barrier towards a lower state. In (a), both diabatic minima (dashed) have the same energy and the barrier arises from electronic coupling of the two diabats. In (b) and (c), the left minimum is shifted to higher energy, such that in (c) the left diabat does not produce a true minimum on the adiabatic potential energy surface (solid red).

In Mn3344 (*S*_1_), we find two low‐energy minima, O4zx4 and O44xy. Both are analogous to the two‐axes crossing minimum (44xy) that is the lowest‐energy minimum of the precatalyst in the same oxidation state. The other five stable minima obtained for the catalyst in the Mn3344 oxidation state (Hx4x4, Oy4x4, Oxz44, Hy4x4, Hz4x4) all feature an apical JT axis. This can be identified as the reason for the slightly higher energy of these five minima (2.0–4.2 kcal/mol). Given the cost of an apical JT axis of 7.5 kcal/mol, we estimate that the cost of a two‐axes crossing in the catalyst is about 5 kcal/mol. Based on these costs, Table S9 shows that there should be seven low‐energy JT arrangements (4zx4, 44xy, xz44, z4x4, y44x4, x4x4, zz44) that should give rise to 14 low‐lying minima for the Mn3344 oxidation state of the catalyst. Five of those would contain a JT axis towards the hydroxide ligand and are energetically unfavorable. From the remaining 9 minima, only seven were found and two minima (H44xy and Ox4x4) could not be optimized, even though none of the five already established rules exclude them. Based on the results of the optimizations, it appears that the barriers for conversion to a lower‐energy minimum (O44xy and Oy4x4, respectively) are so small that the distinct minima vanish. This can be understood from Figure 5, where two diabatic states with constant electronic coupling produce two distinct minima if the minima have similar energies (a and b), but if one of the diabatic curves is shifted to too high energy (or the coupling is increased), only one minimum is obtained (c) for the ground state adiabatic surface.

For Mn3334 (*S*_0_), there are in principle 12 possible minima that do not involve the unfavorable vanadate JT axes (Figures S3 and S4 in the Supporting Information). Out of those, we could optimize four minima, three of which have a two‐axes crossing and an apical JT axis, and one minimum (O4zxy) that has a three‐axis crossing. Rule five is followed in all cases, i. e., no minimum with a hydroxide JT axis was found (compare Table S10 in the Supporting Information). In close analogy to Mn3344, two further minima that are not excluded by any of the proposed rules (Hy4xy and Oxzx4) could not be optimized due to vanishing barriers to lower‐energy minima (Oy4xy and Oyzx4, respectively).

Lastly, for Mn3333 all optimizations converged to the same structure, Oyzxy. This structure exhibits a Mn−OH_2_ bond length of about 3.7 Å, clearly showing signs of ligand dissociation, similar to the observation for the precatalyst in the Mn3333 oxidation state. Although a frequency calculation indicates that with the employed computational parameters this is indeed a minimum on the potential energy surface, it is doubtful whether this distant water ligand would stay associated with the complex in a real solvent environment. Hence, here we prefer not to discuss the Mn3333 structures in more detail.

As no structures with vanadate JT axes were optimized for Mn3334 and Mn3333 due to their large number, we cannot exclude the existance of other minima for these oxidation states. However, based on the findings of the precatalyst, we do not expect that any of those will actually constitute the lowest‐energy minimum for these oxidation states.

To conclude, here we summarize the five heuristic rules about energetically unfavorable JT arrangements in the investigated compounds:

1. A JT axis at the apical Mn atom entails a 10 kcal/mol (7.5 kcal/mol in the catalyst) cost.
2. A two‐axes crossing costs 6 kcal/mol (5 kcal/mol in the catalyst).
3. A vanadate JT axis costs 12 kcal/mol (unknown but possibly similar in the catalyst).
4. A three‐axes crossing costs 19 kcal/mol (roughly 17 kcal/mol in the catalyst).
5. A hydroxide JT axis in the catalyst costs a large amount of energy.

Other specifics of the JT arrangement – like the presence or absence of parallel, antiparallel, or skew arrangements or the position of the water proton – seem to play rather minor roles in the overall energetics. Naturally, these energetic costs carry some uncertainty, due to the limited accuracy of the chosen level of theory, neglect of the low‐spin configurations, anharmonicity, etc. In order to estimate the uncertainty due to the electronic structure level of theory, we performed additional single point calculations at the optimized geometries. It is well known that the inclusion of Hartree–Fock exchange into density functionals can have a strong impact on relative electronic energies, see, e. g., Refs. [56, 57]. Therefore, these additional calculations were performed with B3LYP and CAM‐B3LYP to estimate the influence of exchange. Furthermore, we also inspected the effect of the basis set through BP86/def2‐SVP calculations. As shown in Section S7 and Table S11 in the Supporting Information, only small deviations in the relative free energy differences were found between the different functionals or basis sets, indicating that our findings are robust within the limitations of DFT.[^58^, ^59^, ^60^] Based on these calculations, an apical JT axis costs 9–10 kcal/mol, a two‐axes crossing 6–7 kcal/mol, a vanadate JT axis 12–14 kcal/mol, and a three‐axes crossing about 19–23 kcal/mol in the precatalyst.

These findings provide much needed guidance to explore the catalytic cycle of the MnV WOC and related compounds. The heuristic rules could be used to predict the energetically most favorable JT axis arrangements of intermediates and transition states in the water oxidation mechanism, possibly allowing to exclude a large number of hypothetical structures without performing any quantum chemistry calculations. The finding about water dissociation from Mn3333 might imply that during the catalytic cycle the compound must first be oxidized before it can bind new water molecules. More generally, the rules can help explain the catalytic efficiency and guide the design of new catalysts. For example, assuming that an Mn_4_O_4_ cubane catalyst should support oxidation states from [Mn${}_{4}^{\mathrm{IV}}$ ] to [Mn${}_{4}^{\mathrm{III}}$ ], our rules predict that each Mn atom is required to have at least one coordination axis that can form a low‐cost JT axis. This means that it might not be worthwhile to design a catalyst that is more stabilized by a larger vanadate ligand – if such a vanadate would coordinate with three O atoms on one Mn atom, then that Mn atom could not be reduced to Mn^III^ without forming an energetically costly vanadate JT axis. Hence, the existing MnV WOC seems to be a good compromise between chemical stabilization from the vanadate and chemical reactivity due to the weak bonds to the acetate ligands.

### Comparison to X‐ray structure

**Figure 6:**
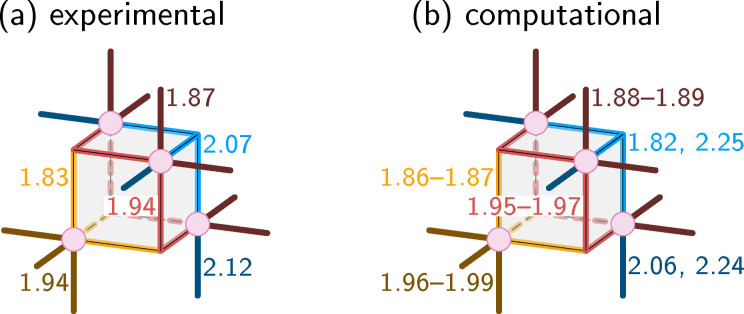
Comparison of experimental (a) and computed (b) bond lengths of the precatalyst in the Mn3344 oxidation state (*S*_1_). All lengths are given in Å. The experimental structure is reported to be *C*_3*v*_ symmetric, whereas the computed structure shows JT distortions that are indicated by giving multiple values.

At this point, a comparison of our findings with the X‐ray crystallographic results of Schwarz et al.^[9]^ is on order. These authors reported the structure of the precatalyst in the Mn3344 oxidation state (*S*_1_) – in the form of (*n*‐Bu_4_N)_3_[Mn_4_O_4_(V_4_O_13_)(OAc)_3_] ⋅ 3H_2_O (CCDC 898055) – to be of ideal *C*_3*v*_ symmetry. The bond lengths found in the crystal structure are shown in Figure 6a. The lengths within the cubane structure differ significantly – the bonds with the apical Mn are the shortest ones (1.83 Å), the bonds with the apical O are the longest ones (2.07 Å), and the other bonds are in between (1.94 Å). The authors argue that the stretched bonds to the apical O atom are due to an attractive electrostatic interaction of that O atom with the vanadate ligand. The crystal structure data also shows that the Mn−O bonds towards the vanadate ligand are short (1.87 Å) and, interestingly, the ones towards the acetates are short at the apical Mn (1.94 Å) and long at the other Mn atoms (2.12 Å). No explanation is given for the disparity of the latter bond lengths. Most interestingly, no JT distortions were observed in the crystal structure, but the authors^[9]^ did not discuss this issue further.

Our results (Figure 6b) mostly agree with the experimental values, except for the JT axes. Bonds not involved in JT axes are 0.01–0.03 Å longer than in the crystal structure, which could be either due to crystal packing/crystal environment effects that are not modeled in the computations, or due to a slight overestimation of the bond length by the employed level of theory. More importantly, while the experimental structure appears to be *C*_3*v*_ symmetric, our optimized structure exhibits a strong JT effect of the acetate axes of two of the non‐apical Mn atoms. The corresponding bond lengths of the Mn^IV^ atom are 1.82 and 2.06 Å, whereas the two Mn^III^ atoms show values of 2.25 and 2.24 Å. In the computed results, the JT effect provides a clear explanation for the fact that the bonds from non‐apical Mn to acetate can be much longer than the ones from the apical Mn to the acetates. The question is now how to resolve this apparent discrepancy between the JT‐distortion‐free crystal structure (implying valence delocalization) and the JT‐distorted computed results (implying valence localization).

**Figure 7:**
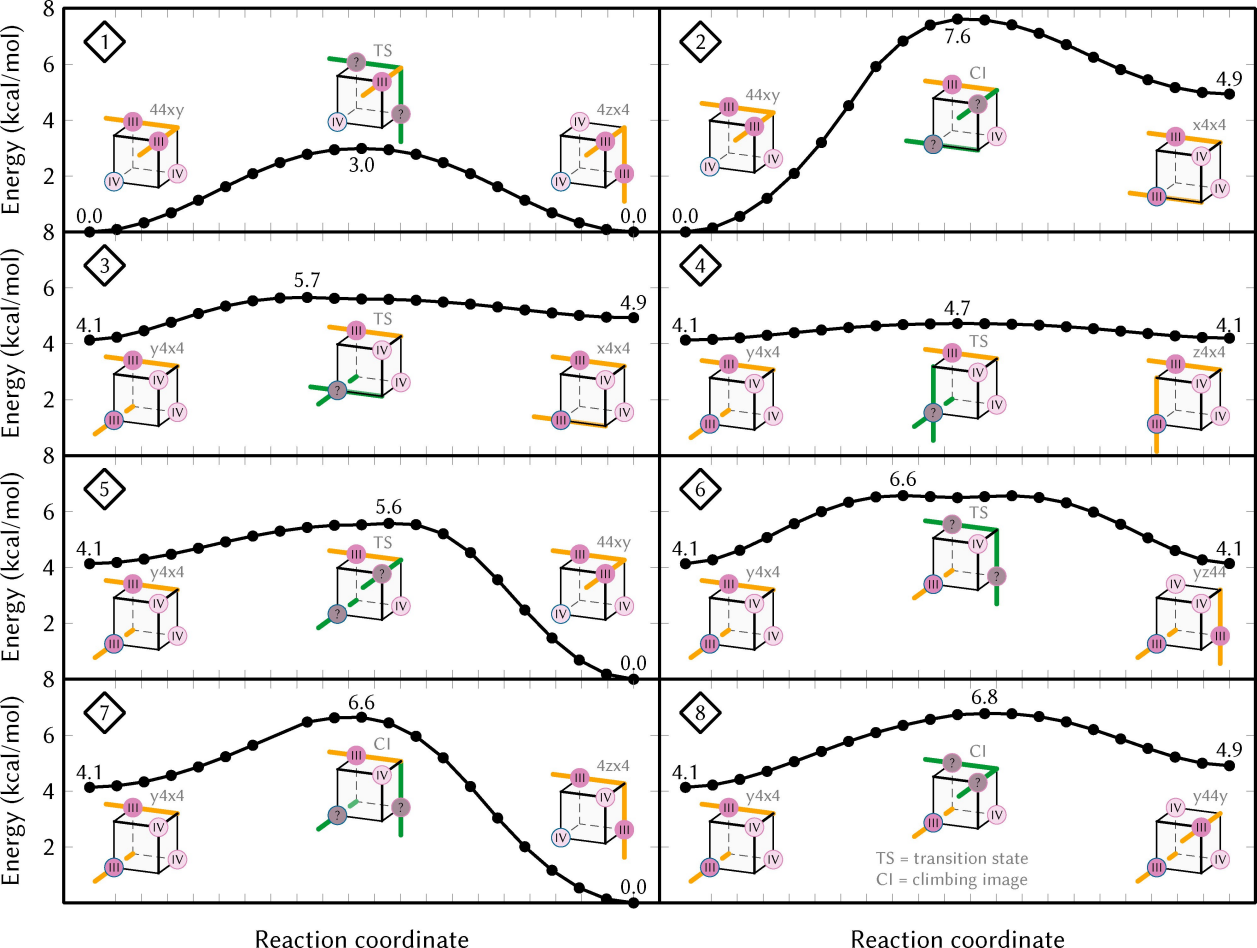
Potential energy surface scans of the eight symmetry‐nonequivalent transition paths of the precatalyst (Mn3344) (see also Figure S6), with energies given in kcal/mol. The drawings indicate the arrangement of the JT axes, with green color denoting the pair of JT axes that contract/expand along the transition path. Note that the scans were performed with linear interpolation in Cartesian coordinates and that some transition states could not be fully optimized. Hence, all barriers in the Figure should be regarded as upper bounds to the true transition state energies. These scans report electronic energies, explaining the small differences to the free energies of the minima in Table 2.

One possible explanation for this discrepancy is dynamic disorder.[^61^, ^62^] This effect describes that in a crystal thermal motion allows the population of different equilibrium positions at different times or in different molecules. An argument for this explanation is the fact that the experimental bond lengths of the non‐apical acetate axes (bond lengths 2.07 and 2.12 Å) are close to the average of the computed bond lengths (2.11 and 2.19 Å), although again the computed bond lengths are slightly too long. However, dynamic disorder can only occur if the conversion barriers between the different JT arrangements are small enough compared to the temperature at which the crystal structure is recorded. For this reason, we computed all possible interconversion barriers between the stable JT arrangements of the precatalyst in the Mn3344 oxidation state. The 39 possible paths and the eight classes of symmetry‐nonequivalent paths are shown in Figure S6 in the Supporting Information, while Figure 7 presents potential energy surface scans for all eight classes. Note that all barriers should be regarded as upper bounds for the true transition barriers, due to the linear interpolation used for generating the scans. Based on the energetics, the most relevant interconversion path is the one that connects the lowest‐energy minima of this structure (i. e., two different two‐axes crossing minima, 44xy, 4zx4, and 4z4y). We find a potential energy barrier of only 3 kcal/mol, which according to Eyring's equation corresponds to a life time of each JT arrangement of only 25 ps at 300 K. At the temperature used to record the crystal structure,^[9]^ 150 K, the life time is still only about 7.5 ns, much shorter than the data collection time. There are even theoretical works^[63]^ showing that tunneling can contribute to JT interconversion dynamics for such small barriers (about 2 kcal/mol in the cited work).

Hence, our computations strongly suggest that the MnV WOC undergoes very rapid interconversion between localized minima with different JT arrangements, and that the reported crystal structure only observes the averages of non‐JT and JT bond lengths. In the widely used classification scheme for mixed‐valence transition metal complexes by Robin and Day,[^64^, ^65^] this corresponds to a class II intermediate localized situation. As it has been noted before,^[66]^ computational results on such systems require a fine balance between dynamic and static correlation effects. Here, we use a functional that is based on the generalized gradient approximation (BP86), which tends to suffer somewhat from self‐interaction errors and thus should produce a too delocalized electronic structure.[^58^, ^67^] This is interesting, because for the MnV WOC we find an electronic structure that is more localized than the experiment even with a functional that should produce too much delocalization. Hence, we assume that the disagreement between experiment and computations is not due to the limitations of our level of theory. We note that it has been discussed before^[55]^ that DFT can correctly describe valence localization in multi‐center transition metal complexes even though the self‐interaction error of many DFT functions in principle favors delocalized electronic structures. As limitations of the level of theory are probably not responsible for the mentioned discrepancy, an alternative explanation could be the different effect of the surroundings – in solution a localized electronic structure is formed, whereas in the crystal a delocalized one arises. Nevertheless, we suggest that it might be worthwhile to revisit the delocalization–localization issue in this complex (optimally for multiple oxidation states) from the experimental side, to scrutinize our finding that the electronic structure of the complex is valence‐localized and the structure is disordered between multiple JT‐distorted minima.

The very low interconversion barriers that we found in our work for the solvated MnV WOC can also help to explain the distinguishing feature of the optimal ligand exchange pathway found for the catalyst activation mechanism.^[35]^ As shown above, a JT axis of Mn^III^ tends to be weaker and more easily undergoes ligand dissociation than a coordination axis of Mn^IV^. The low barriers in Figure 7 can then be exploited to easily switch the location of the most reactive coordination site on the cubane – like first weakening a bond to the acetate ligand, exchanging this ligand with water from the solution, then moving the JT axis to another Mn center and exchanging a second ligand.^[35]^

### Comparison to other complexes

It is also interesting to compare our findings to previously reported JT distortions in other Mn_4_O_4_ complexes. As mentioned above, the first such complex had six diphenylphosphinate ligands[^6^, ^19^, ^68^] and was claimed to not exhibit JT distortions in the Mn3344 oxidation state, although it was speculated^[19]^ that this might be due to disorder. Very preliminary optimizations of this complex by us (using the same method described above) always lead to a geometry with two skew‐oriented JT axes.

The same complex, but in the Mn3444 oxidation state, was also claimed to not show JT distortions.[^15^, ^68^] However, when changing to six bis(p‐methoxyphenyl)phosphinate ligands,[^7^, ^68^] the Mn3444 oxidation state showed one JT axis and the Mn3344 oxidation state showed two antiparallel JT axes (compare Figure 4). The same antiparallel JT arrangement for Mn3344 was reported for a complex with six di(*t*‐butyl)phosphate ligands^[69]^ and another complex with six dimethylarsinate ligands.^[23]^ This might indicate that in homoleptic Mn_4_O_4_ cubanes, antiparallel JT arrangements might be energetically favorable. However, for the complex with diphenylphosphinate ligands, we were not able to optimize an antiparallel arrangement. In the heteroleptic MnV WOC, all antiparallel arrangements involve a vanadate JT axis (compare Figure 3), and hence are energetically not favorable.

The Agapie group has also reported a Mn_4_O_4_ cubane capped with a hexadentate ligand (coordinating through pyridine and hydroxyl groups) and either three diphenylphosphinate ligands or three acetate ligands,[^10^, ^24^, ^25^] where the latter is formally quite similar to the MnV WOC. The DFT calculations of these authors^[25]^ seem to indicate a JT axis at non‐apical Mn atom for Mn3444. For Mn3344, the authors presented two skew‐oriented axes (Figure S3 in Ref. [25]) – both on non‐apical Mn atoms. This is similar to the behaviour of our MnV WOC, which tends to avoid apical JT axes, due to their high energetic cost (first rule).

## Conclusion

We reported a comprehensive computational study of the Jahn‐Teller effect on the energetics of different oxidation and tautomeric states of the manganese‐oxo vanadate catalyst [(Mn_4_O_4_)(V_4_O_13_)(OAc)_3_]^*n*−^ and its activated species [(Mn_4_O_4_)(V_4_O_13_)(OAc)_2_(H_2_O)(OH)]^*n*−^. By optimizing all combinatorially possible arrangements of the Jahn‐Teller axes at the Mn^III^ atoms, we found that for each oxidation state, only few Jahn‐Teller arrangements are energetically stable, i. e., there are only one or two arrangements for each oxidation state at thermal equilibrium.

By correlating the energetics of the different optimized minima with the main structural features, we derived a set of heuristic rules that can be utilized to estimate the relative energies of different Jahn‐Teller arrangements. The first rule states that a Jahn‐Teller axis at the apical Mn atom, the one bonded to all three acetate ligands, is energetically less favorable (by about 10 kcal/mol) than a Jahn‐Teller axis at any of the three other Mn atoms. Rule two states that the crossing of two Jahn‐Teller axes brings an energetic cost (about 6 kcal/mol). Third, Jahn‐Teller axes pointing towards the vanadate O atoms are very unfavorable (about 12 kcal/mol). The fourth rule associates a very high cost (about 19 kcal/mol) to the crossing of three Jahn‐Teller axes. Finally, in the activated complex, also Jahn‐Teller axes towards the hydroxide ligand entail a high energetic cost. Otherwise, the relative orientation of the Jahn‐Teller axes (e. g., parallel, antiparallel, skew orientations) seem to have little effect on the energy of the molecule. These rules likely derive from the electrostatic interaction of the additional electron of Mn^III^ with the different ligands, as well as the abilities of the ligands for *σ* and *π* donation. In particular, the vanadate ligand binds rather tightly to the Mn atoms, stabilizing the entire cluster but not participating in the formation of the Jahn‐Teller axes.

We have also discussed the issue of a localized versus delocalized mixed‐valence electronic structure for the complex. Based on all results that we have collected, at the various minima the MnV WOC always exhibits clearly localized +III and +IV oxidation states, stabilized by the formation of the Jahn‐Teller axes. The finding of clearly localized oxidation states seems at odds with some previous X‐ray structures of the MnV WOC^[9]^ and similar tetramanganese oxo cubanes[^6^, ^15^, ^19^, ^68^] that reported the absence of clear Jahn‐Teller distortions despite the formal presence of Mn^III^. We showed that this is likely due to the presence of very small barriers between different Jahn‐Teller arrangements, enabling dynamic disorder to take place even at low temperatures.

Although quantitative energetic costs will depend on the molecule at hand (and will also vary depending on the level of theory), these rules will help predicting general trends in the structure of transition metal oxo cubanes (and other clusters/polyoxometalates) as well as explaining their reactivity and properties. The availability of different Jahn‐Teller minima and the involved low barriers for interconversion among them can facilitate important reaction steps for oxygen‐evolving catalysts, such as the ligand exchange observed during activation^[35]^ of the here studied MnV WOC. Thus, we expect that the presented findings will enable the directed search for even more efficient catalysts.

## Supporting Information

Depictions and lists of all possible JT arrangements and their symmetry relations. Depictions of optimized and hypothetical minima. Bond lengths from pre‐optimizations and full optimizations. Molecular orbitals. Population analysis. Predicted relative energies of all possible JT arrangements based on the five presented rules. Error estimation for heuristic rules. Overview over JT conversion pathways. Cartesian coordinates (xyz format) for all stable minima. Absolute energies for all stable minima and Gaussian 16 input.

## Conflict of interest

The authors declare no conflict of interest.

## Supporting information

As a service to our authors and readers, this journal provides supporting information supplied by the authors. Such materials are peer reviewed and may be re‐organized for online delivery, but are not copy‐edited or typeset. Technical support issues arising from supporting information (other than missing files) should be addressed to the authors.

Supporting Information
