# Supplementary material for: Jahn‐Teller Effects in a Vanadate‐Stabilized Manganese‐Oxo Cubane Water Oxidation Catalyst
Source: Chemistry. 2021 Nov 5;27(68):17066–77. doi: 10.1002/chem.202102539 (PMC9298120; doi:10.1002/chem.202102539)
Supplement: Supplementary file 1 — Supporting Information [file CHEM-27-17066-s001.pdf]

Chemistry–A European Journal

Supporting Information

Jahn-Teller Effects in a Vanadate-Stabilized Manganese-Oxo Cubane Water Oxidation Catalyst

Sebastian Mai,* Marcus Holzer, Anastasia Andreeva, and Leticia González*

Contents

S1 Symmetry 2

S2 Depictions of optimized and hypothetical minima 5

S3 Pre-optimization and optimization results 8

S4 Molecular orbitals 11

S5 Population analysis 12

S6 Predicted energies 13

S7 Error estimation for heuristic rules 16

S8 Transition paths between Jahn–Teller Arrangements 17

S9 Absolute energies of reported geometries 19

S10 Input parameters for Gaussian 16 20

S1 Symmetry

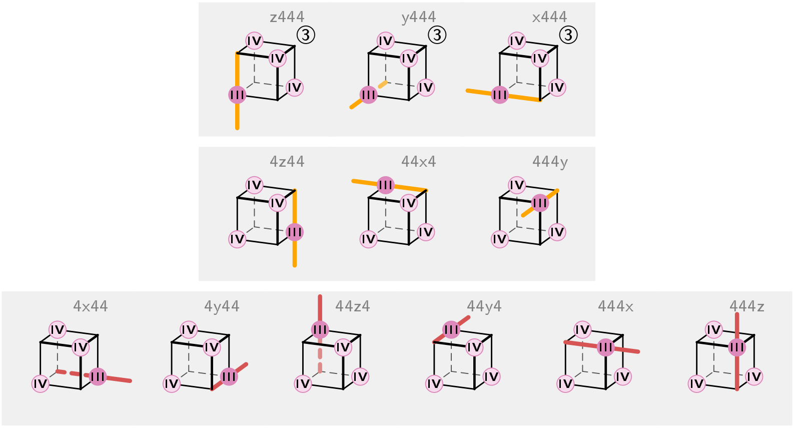

Figure S1: Overview over all possible Jahn-Teller axis arrangements of the Mn3444 oxidation state of the precatalyst. Each row shows the three or six symmetry-equivalent arrangements: (top) Jahn-Teller axis is on the apical Mn atom, (middle) Jahn-Teller axis is on a non-apical Mn and points towards an acetate ligand, (bottom) Jahn-Teller axis is on a non-apical Mn and points towards the vanadate ligand.

Table S1: Overview over all JT labels and their symmetry equivalences in the C_{3v} point group. Greyed out labels are those that already appeared in the table because several symmetry operations produce the same label. In this way, the number of non-greyed labels is 256, the total number of possible JT arrangements. h is the degeneracy, given as the number of distinct labels on each line.

	$\hat{E}$	$\hat{C}_3$	$\hat{C}_3^2$	$\hat{\sigma}_{AB}$	$\hat{\sigma}_{AC}$	$\hat{\sigma}_{AD}$	h
Mn4444	4444	4444	4444	4444	4444	4444	1
Mn3444	z444	x444	y444	z444	y444	x444	3
	4z44	44x4	444y	4z44	44x4	444y	3
	4x44	44y4	444z	4y44	444x	44z4	6
Mn3344	zz44	x4x4	y44y	zz44	y44y	x4x4	3
	zy44	y44x	x4z4	zx44	x4y4	y44z	6
	yz44	x44y	z4x4	xz44	y4x4	z44y	6
	yy44	x44x	z4z4	xx44	y4y4	z44z	6
	xy44	y4z4	z44x	yx44	x44z	z4y4	6
	44xx	4y4y	4zz4	44yy	4xx4	4z4z	6
	44xy	4z4y	4zx4	44xy	4zx4	4z4y	3
	44xz	4x4y	4zy4	44zy	4yx4	4z4x	6
	44yx	4y4z	4xz4	44yx	4xz4	4y4z	3
	44yz	4x4z	4xy4	44zx	4yz4	4y4x	6
	44zz	4x4x	4yy4	44zz	4yy4	4x4x	3
Mn3334	zzz4	x4xx	yy4y	zz4z	y4yy	xxx4	6
	zzx4	x4xy	yz4y	zz4y	y4xy	xzx4	6
	zzy4	x4xz	yx4y	zz4x	y4zy	xyx4	6
	zxz4	x4yx	yy4z	zy4z	y4yx	xxz4	6
	zxx4	x4yy	yz4z	zy4y	y4xx	xzz4	6
	zxy4	x4yz	yx4z	zy4x	y4zx	xyz4	6
	zyz4	x4zx	yy4x	zx4z	y4yz	xxy4	6
	zyx4	x4zy	yz4x	zx4y	y4xz	xzy4	6
	zyy4	x4zz	yx4x	zx4x	y4zz	xyy4	6
	yzz4	z4xx	xy4y	xz4z	z4yy	yxx4	6
	yzx4	z4xy	xz4y	xz4y	z4xy	yzx4	3
	yzy4	z4xz	xx4y	xz4x	z4zy	yyx4	6
	yxz4	z4yx	xy4z	xy4z	z4yx	yxz4	3
	yxy4	z4yz	xx4z	xy4x	z4zx	yyz4	6
	yyy4	z4zz	xx4x	xx4x	z4zz	yyy4	3
	4zzz	4xxx	4yyy	4zzz	4yyy	4xxx	3
	4zzx	4yxx	4yzy	4zyz	4xyy	4xxz	6
	4zzy	4zxx	4yxy	4zxz	4zyy	4xxy	6
	4zxy	4zxy	4zxy	4zxy	4zxy	4zxy	1
	4zyx	4yxz	4xzy	4zyx	4xzy	4yxz	3
	4xzz	4xyx	4yyz	4yzz	4yyx	4xzx	6
	4xyz	4xyz	4xyz	4yzx	4yzx	4yzx	2
Mn3333	zzzz	xxxx	yyyy	zzzz	yyyy	xxxx	3
	zzzx	xyxx	yyzy	zzyz	yxyy	xxxz	6
	zzzy	xzxx	yyxy	zzxz	yzyy	xxxy	6
	zzxx	xyxy	yzzy	zzyy	yxxy	xzxz	6
	zzxy	xzxy	yzxy	zzxy	yzxy	xzxy	3
	zzyx	xyxz	yxzy	zzyx	yxzy	xyxz	3
	zxzz	xxyx	yyyz	zyzz	yyyx	xxzx	6
	zxzx	xyyx	yyzz	zyyz	yxyx	xxzz	6
	zxzy	xzyx	yyxz	zyxz	yzyx	xxzy	6
	zxxz	xxyy	yzyz	zyzy	yyxx	xzzx	6
	zxxx	xyyy	yzzz	zyyy	yxxx	xzzz	6
	zxxy	xzyy	yzxz	zyxy	yzxx	xzzy	6
	zxyz	xxyz	yxyz	zyzx	yyzx	xyzx	6
	zxyx	xyyz	yxzz	zyyx	yxzx	xyzz	6
	zxyy	xzyz	yxxz	zyxx	yzzx	xyzy	6

Table S2: Overview over all JT labels and their symmetry equivalences in the C_s point group. Greyed out labels are those that already appeared for the same pair because both symmetry operations produce the same label. In this way, the number of non-greyed labels is 256, the total number of possible JT arrangements. h is the degeneracy, given as the number of distinct labels in each group.

	$\hat{E}$	$\hat{\sigma}_{AB}$	h	$\hat{E}$	$\hat{\sigma}_{AB}$	h	$\hat{E}$	$\hat{\sigma}_{AB}$	h
Mn4444	4444	4444	1						
Mn3444	z444	z444	1	x444	y444	2	4z44	4z44	1
	4x44	4y44	2	44z4	444z	2	44x4	444y	2
	44y4	444x	2						
Mn3344	zz44	zz44	1	zx44	zy44	2	xz44	yz44	2
	xx44	yy44	2	xy44	yx44	2	z4z4	z44z	2
	z4x4	z44y	2	z4y4	z44x	2	x4z4	y44z	2
	x4x4	y44y	2	x4y4	y44x	2	y4z4	x44z	2
	y4x4	x44y	2	y4y4	x44x	2	4zz4	4z4z	2
	4zx4	4z4y	2	4zy4	4z4x	2	4xz4	4y4z	2
	4xx4	4y4y	2	4xy4	4y4x	2	4yz4	4x4z	2
	4yx4	4x4y	2	4yy4	4x4x	2	44zz	44zz	1
	44zx	44yz	2	44zy	44xz	2	44xx	44yy	2
	44xy	44xy	1	44yx	44yx	1			
Mn3334	zzz4	zz4z	2	zzx4	zz4y	2	zzy4	zz4x	2
	zxz4	zy4z	2	zxx4	zy4y	2	zxy4	zy4x	2
	zyz4	zx4z	2	zyx4	zx4y	2	zyy4	zx4x	2
	xzz4	yz4z	2	xzx4	yz4y	2	xzy4	yz4x	2
	xxz4	yy4z	2	xxx4	yy4y	2	xxy4	yy4x	2
	xyz4	yx4z	2	xyx4	yx4y	2	xyy4	yx4x	2
	yzz4	xz4z	2	yzx4	xz4y	2	yzy4	xz4x	2
	yxz4	xy4z	2	yxx4	xy4y	2	yxy4	xy4x	2
	yyz4	xx4z	2	yyx4	xx4y	2	yyy4	xx4x	2
	z4zz	z4zz	1	z4zx	z4yz	2	z4zy	z4xz	2
	z4xx	z4yy	2	z4xy	z4xy	1	z4yx	z4yx	1
	x4zz	y4zz	2	x4zx	y4yz	2	x4zy	y4xz	2
	x4xz	y4zy	2	x4xx	y4yy	2	x4xy	y4xy	2
	x4yz	y4zx	2	x4yx	y4yx	2	x4yy	y4xx	2
	4zzz	4zzz	1	4zzx	4zyz	2	4zzy	4zxz	2
	4zxx	4zyy	2	4zxy	4zxy	1	4zyx	4zyx	1
	4xzz	4yzz	2	4xzx	4yyz	2	4xzy	4yxz	2
	4xxz	4yzy	2	4xxx	4yyy	2	4xxy	4yxy	2
	4xyz	4yzx	2	4xyx	4yyx	2	4xyy	4yxx	2
Mn3333	zzzz	zzzz	1	zzzx	zzyz	2	zzzy	zzxz	2
	zzxx	zzyy	2	zzxy	zzxy	1	zzyx	zzyx	1
	zxzz	zyzz	2	zxzx	zyyz	2	zxzy	zyxz	2
	zxxz	zyzy	2	zxxx	zyyy	2	zxxy	zyxy	2
	zxyz	zyzx	2	zxyx	zyyx	2	zxyy	zyxx	2
	xzzz	yzzz	2	xzzx	yzyz	2	xzzy	yzxz	2
	xzxz	yzzy	2	xzxx	yzyy	2	xzxy	yzxy	2
	xzyz	yzzx	2	xzyx	yzyx	2	xzyy	yzxx	2
	xxzz	yyzz	2	xxzx	yyyz	2	xxzy	yyxz	2
	xxxz	yyzy	2	xxxx	yyyy	2	xxxy	yyxy	2
	xxyz	yyzx	2	xxyx	yyyx	2	xxyy	yyxx	2
	xyzz	yxzz	2	xyzx	yxyz	2	xyzy	yxxz	2
	xyxz	yxzy	2	xyxx	yxyy	2	xyxy	yxxy	2
	xyyz	yxzx	2	xyyx	yxyx	2	xyyy	yxxx	2

S2 Depictions of optimized and hypothetical minima

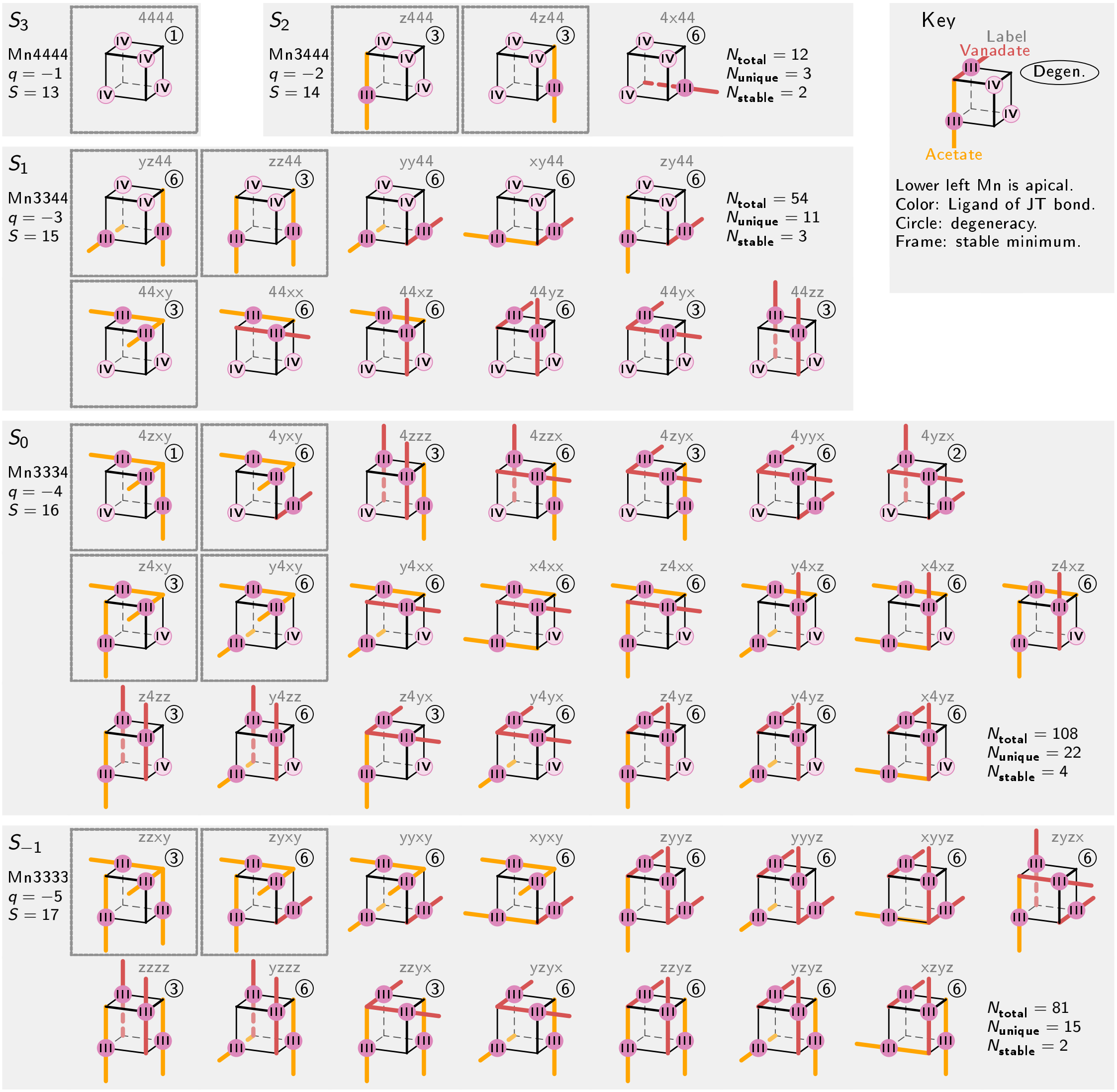

Figure S2: Overview over all possible, unique Jahn-Teller axis arrangements of the precatalyst. Each block corresponds to one overall oxidation state (S_3, S_2, ...; indicated by charge q and spin S). Each cube is oriented in the same way, with the apical Mn atom in the lower-left corner. Jahn-Teller axes are indicated by colored, thick lines, with the color indicating the type of bonded ligand. The degeneracy of the arrangement and a label are also given. A frame indicates which arrangements correspond to minima.

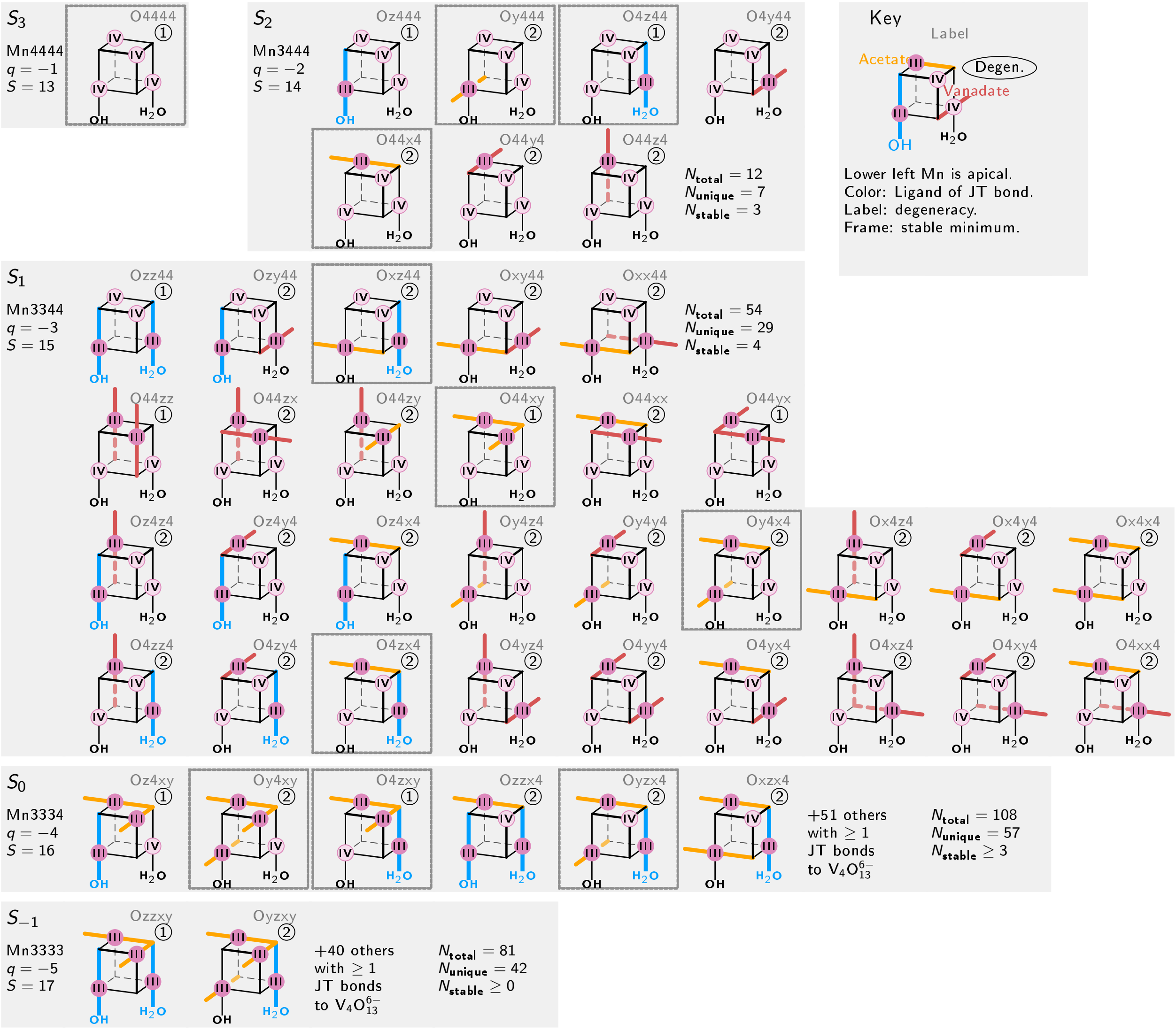

Figure S3: As Figure S2, but for the catalyst with hydroxide bonded to the apical Mn atom. For the S_0 and S_{-1} oxidation states, only arrangements with zero Jahn-Teller axes pointing towards the vanadate ligand were considered.

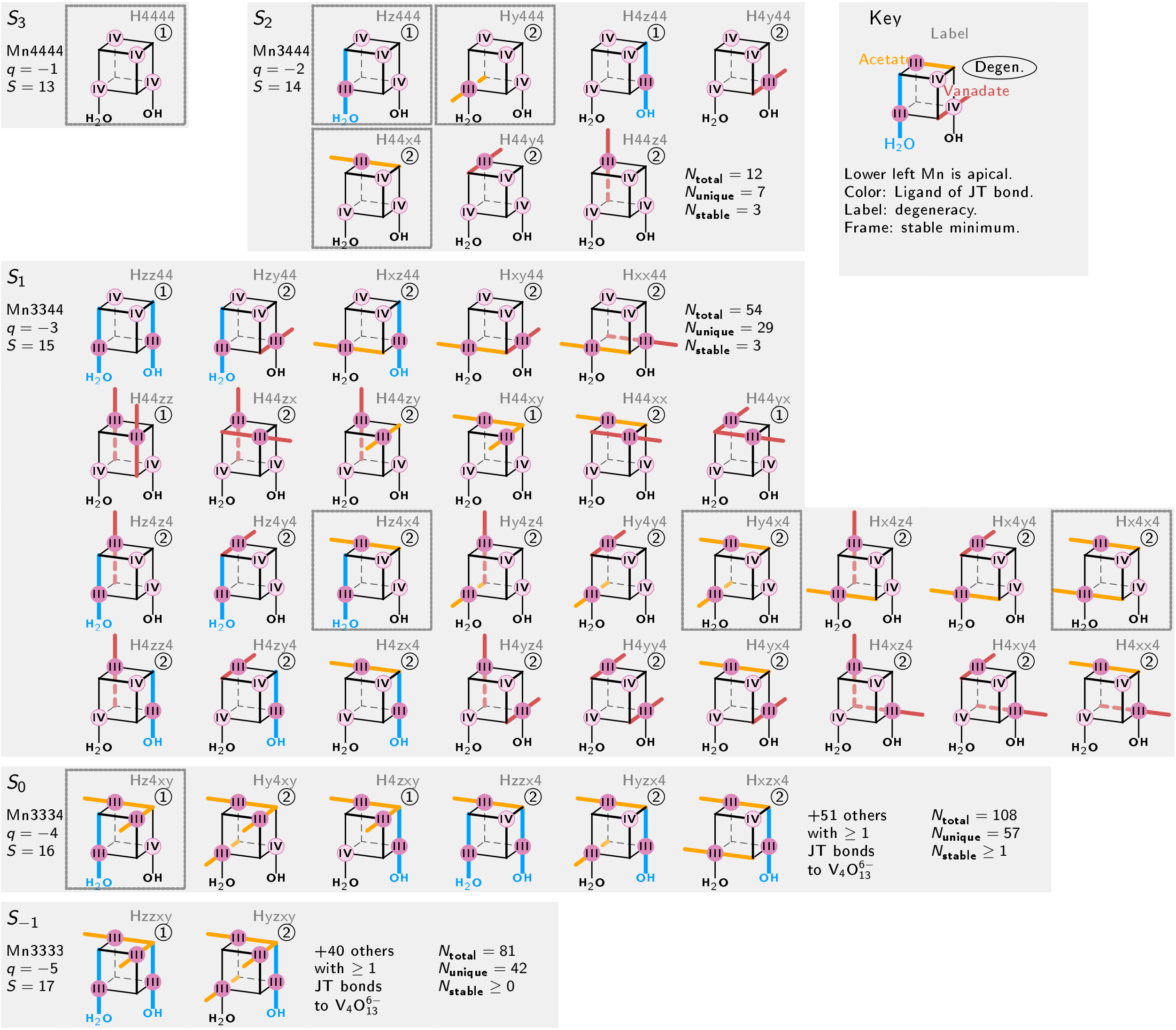

Figure S4: As Figure S3, but for the catalyst with water bonded to the apical Mn atom.

S3 Pre-optimization and optimization results

Table S3: Average bond lengths (Å) of the 12 Mn coordination axes before (left) and after (right) full optimization. Values above 2.13Å are highlighted in red, as these indicate a JT axis. The labels on the right are not necessarily corresponding directly to the shown bond lengths, but give the symmetry-equivalent label from Figure S2 (see also Table S1).

— After pre-optimization —													— After full optimization —												
Mn atom A			Mn atom B			Mn atom C			Mn atom D			Label	Mn atom A			Mn atom B			Mn atom C			Mn atom D			Label
z	*x*	*y*	*z*	*x*	*y*	*z*	*x*	*y*	*z*	*x*	*y*		*z*	*x*	*y*	*z*	*x*	*y*	*z*	*x*	*y*	*z*	*x*	*y*	
												— Mn4444 —													
1.90	1.90	1.90	1.95	1.88	1.88	1.88	1.95	1.88	1.88	1.88	1.95	4444	1.91	1.91	1.91	1.96	1.89	1.89	1.89	1.96	1.89	1.89	1.89	1.96	4444
												— Mn3444 —													
2.23	1.94	1.94	1.94	1.89	1.88	1.90	1.95	1.87	1.90	1.87	1.95	z444	2.20	1.95	1.95	1.94	1.90	1.90	1.91	1.95	1.88	1.91	1.88	1.95	z444
1.90	1.91	1.92	2.23	1.92	1.91	1.90	1.96	1.88	1.89	1.88	1.96	4z44	1.90	1.92	1.91	2.25	1.92	1.92	1.91	1.95	1.89	1.91	1.89	1.95	4z44
1.90	1.92	1.90	2.15	2.13	1.88	1.88	1.95	1.89	1.90	1.88	1.94	4x44	1.90	1.92	1.91	2.25	1.92	1.92	1.91	1.95	1.89	1.91	1.89	1.95	4z44
												— Mn3344 —													
2.23	1.94	1.94	2.23	1.92	1.92	1.91	1.95	1.88	1.91	1.88	1.95	zz44	2.17	1.95	1.95	2.24	1.93	1.93	1.92	1.95	1.89	1.93	1.89	1.95	zz44
2.23	1.91	1.98	2.10	1.88	2.13	1.91	1.95	1.87	1.90	1.88	1.96	zy44	2.17	1.95	1.95	2.24	1.93	1.93	1.92	1.95	1.89	1.93	1.89	1.95	zz44
1.94	2.23	1.95	2.23	1.96	1.90	1.90	1.95	1.89	1.88	1.91	1.97	yz44	1.93	2.19	1.96	2.27	1.95	1.90	1.91	1.94	1.90	1.89	1.91	1.95	yz44
1.94	1.93	2.23	2.16	1.87	2.13	1.89	1.95	1.90	1.89	1.89	1.94	yy44	1.93	2.19	1.96	2.27	1.95	1.90	1.91	1.94	1.90	1.89	1.91	1.95	yz44
1.92	2.23	1.97	2.18	1.89	2.13	1.90	1.93	1.89	1.87	1.91	1.95	xy44	1.93	2.19	1.96	2.27	1.95	1.90	1.91	1.94	1.90	1.89	1.91	1.95	yz44
1.94	1.90	1.90	1.95	1.89	1.89	2.13	2.12	1.88	2.13	1.88	2.12	44zz	1.93	1.91	1.91	1.94	1.91	1.91	1.93	2.25	1.93	1.93	1.93	2.25	44xy
1.92	1.92	1.90	1.95	1.88	1.90	2.13	2.10	1.87	1.87	2.13	2.12	44yz	1.93	1.91	1.91	1.94	1.91	1.91	1.93	2.25	1.93	1.93	1.93	2.25	44xy
1.90	1.92	1.92	1.94	1.90	1.90	1.88	2.16	2.13	1.88	2.13	2.16	44yx	1.93	1.91	1.91	1.94	1.91	1.91	1.93	2.25	1.93	1.93	1.93	2.25	44xy
1.93	1.89	1.91	1.95	1.90	1.88	1.91	2.23	1.93	2.13	1.88	2.08	44xz	1.93	1.91	1.91	1.94	1.91	1.91	1.93	2.25	1.93	1.93	1.93	2.25	44xy
1.92	1.90	1.90	1.93	1.90	1.90	1.93	2.23	1.92	1.93	1.92	2.23	44xy	1.93	1.91	1.91	1.94	1.91	1.91	1.93	2.25	1.93	1.93	1.93	2.25	44xy
1.91	1.91	1.90	1.94	1.89	1.90	1.95	2.23	1.89	1.88	2.13	2.10	44xx	1.93	1.91	1.91	1.94	1.91	1.91	1.93	2.25	1.93	1.93	1.93	2.25	44xy
												— Mn3334 —													
2.23	1.93	1.93	1.94	1.89	1.90	2.13	2.15	1.88	2.13	1.88	2.15	z4zz	2.22	1.94	1.94	1.93	1.92	1.92	1.97	2.27	1.91	1.97	1.91	2.27	z4xy
2.23	1.91	1.98	1.94	1.90	1.89	1.89	2.19	2.13	2.13	1.86	2.13	z4yz	2.22	1.94	1.94	1.93	1.92	1.92	1.97	2.27	1.91	1.97	1.91	2.27	z4xy
2.23	1.92	1.95	1.93	1.90	1.89	1.94	2.23	1.91	2.13	1.87	2.13	z4xz	2.22	1.94	1.94	1.93	1.92	1.92	1.97	2.27	1.91	1.97	1.91	2.27	z4xy
2.23	1.93	1.94	1.93	1.90	1.90	1.96	2.23	1.90	1.96	1.90	2.23	z4xy	2.22	1.94	1.94	1.93	1.92	1.92	1.97	2.27	1.91	1.97	1.91	2.27	z4xy
2.23	1.96	1.92	1.93	1.90	1.91	1.99	2.23	1.88	1.89	2.13	2.20	z4xx	2.22	1.94	1.94	1.93	1.92	1.92	1.97	2.27	1.91	1.97	1.91	2.27	z4xy
1.98	1.91	2.23	1.95	1.89	1.90	1.89	2.23	1.96	2.13	1.89	2.04	y4xz	2.22	1.94	1.94	1.93	1.92	1.92	1.97	2.27	1.91	1.97	1.91	2.27	z4xy
1.93	1.89	1.94	2.04	1.89	2.13	1.92	2.23	1.92	2.13	1.90	2.04	4zyx	1.94	1.94	2.22	2.27	1.91	1.97	1.91	2.27	1.97	1.92	1.92	1.93	z4xy
1.92	1.94	1.90	2.07	2.13	1.90	2.13	2.07	1.88	1.88	2.13	2.11	4yyx	1.94	2.22	1.94	2.27	1.97	1.91	1.92	1.93	1.92	1.91	1.97	2.27	z4xy
2.23	1.99	1.92	2.25	1.92	1.91	1.89	1.92	2.13	1.90	2.13	2.21	z4yx	2.17	1.98	1.93	2.25	1.94	1.92	1.93	1.95	1.91	1.95	1.91	2.29	y4xy
2.02	1.90	2.23	1.96	1.88	1.91	2.13	2.19	1.89	2.13	1.88	2.07	y4zz	1.98	1.93	2.17	1.95	1.91	1.93	1.91	2.29	1.95	1.94	1.92	2.25	y4xy
1.96	1.92	2.23	1.96	1.89	1.89	1.87	2.16	2.13	2.13	1.88	2.06	y4yz	1.98	1.93	2.17	1.95	1.91	1.93	1.91	2.29	1.95	1.94	1.92	2.25	y4xy
1.91	1.99	2.23	1.95	1.89	1.91	1.87	2.19	2.13	1.88	2.13	2.13	y4yx	1.98	1.93	2.17	1.95	1.91	1.93	1.91	2.29	1.95	1.94	1.92	2.25	y4xy
1.96	2.23	1.92	1.94	1.91	1.89	1.94	2.23	1.91	1.90	1.95	2.23	y4xy	1.98	2.17	1.93	1.95	1.93	1.91	1.94	2.25	1.92	1.91	1.95	2.29	y4xy
1.93	1.97	2.23	1.95	1.88	1.92	1.92	2.23	1.92	1.88	2.13	2.10	y4xx	1.98	1.93	2.17	1.95	1.91	1.93	1.91	2.29	1.95	1.94	1.92	2.25	y4xy
1.94	2.23	1.96	1.96	1.92	1.87	1.88	2.09	2.13	2.13	1.89	2.15	x4yz	1.98	2.17	1.93	1.95	1.93	1.91	1.94	2.25	1.92	1.91	1.95	2.29	y4xy
1.98	2.23	1.92	1.95	1.92	1.88	1.92	2.23	1.92	2.13	1.89	2.16	x4xz	1.98	2.17	1.93	1.95	1.93	1.91	1.94	2.25	1.92	1.91	1.95	2.29	y4xy
1.94	2.23	1.94	1.95	1.90	1.89	1.95	2.23	1.90	1.87	2.13	2.15	x4xx	1.98	2.17	1.93	1.95	1.93	1.91	1.94	2.25	1.92	1.91	1.95	2.29	y4xy
1.91	1.91	1.91	2.23	1.92	1.92	1.92	2.23	1.92	1.92	1.92	2.23	4zxy	1.92	1.92	1.92	2.24	1.93	1.94	1.93	2.24	1.93	1.93	1.93	2.25	4zxy
1.92	1.92	1.92	2.07	1.88	2.13	2.13	2.08	1.88	1.88	2.13	2.07	4yzx	1.92	1.92	1.92	2.24	1.93	1.94	1.93	2.24	1.93	1.93	1.93	2.25	4zxy
1.91	1.93	1.91	2.06	2.13	1.89	1.92	2.23	1.92	1.89	2.13	2.06	4zzz	1.94	1.93	1.91	1.96	2.19	1.94	1.91	2.32	1.94	1.94	1.92	2.27	4yxy
1.92	1.91	1.92	2.07	2.13	1.88	1.89	2.23	1.95	2.13	1.89	2.03	4zzx	1.94	1.93	1.91	1.96	2.19	1.94	1.91	2.32	1.94	1.94	1.92	2.27	4yxy
1.92	1.90	1.92	1.99	1.91	2.13	1.94	2.23	1.91	1.90	1.94	2.23	4yxy	1.94	1.91	1.93	1.96	1.94	2.19	1.93	2.28	1.93	1.91	1.95	2.31	4yxy
												— Mn3333 —													
1.94	2.23	1.94	2.17	2.13	1.88	1.92	2.23	1.92	1.88	2.13	2.18	zzzz	1.95	2.21	1.95	2.32	1.95	1.91	1.93	2.27	1.93	1.91	1.96	2.30	zzxy
1.97	2.23	1.93	2.17	2.13	1.87	1.90	2.23	1.94	2.13	1.89	2.15	zzyz	1.95	2.21	1.95	2.30	1.96	1.91	1.93	2.27	1.93	1.91	1.95	2.32	zzxy
1.95	2.23	1.96	2.24	1.89	2.13	1.92	2.23	1.92	2.13	1.89	2.23	zzyx	1.95	2.22	1.95	2.30	1.96	1.91	1.93	2.28	1.93	1.91	1.96	2.31	zzxy
2.23	1.94	1.94	2.23	1.92	1.92	1.95	2.23	1.90	1.95	1.90	2.23	zzxy	2.21	1.95	1.95	2.27	1.93	1.93	1.96	2.30	1.91	1.95	1.91	2.32	zzxy
1.96	1.93	2.23	2.16	1.87	2.13	1.91	2.23	1.94	1.92	1.93	2.23	yyxy	1.95	1.95	2.21	2.30	1.91	1.96	1.91	2.32	1.95	1.93	1.93	2.27	zzxy
1.93	2.23	1.96	2.19	1.89	2.13	1.93	2.23	1.91	1.89	1.96	2.23	xyxy	1.95	2.21	1.95	2.30	1.96	1.91	1.93	2.27	1.93	1.91	1.95	2.32	zzxy
2.23	1.95	1.95	2.01	1.89	2.13	2.13	2.15	1.87	1.89	2.13	2.18	zyzx	2.31	1.95	1.96	1.95	1.93	2.31	1.96	2.29	1.91	1.93	1.92	3.10	zyxy
2.23	2.02	1.90	1.97	2.13	1.92	2.13	2.15	1.86	1.90	2.13	2.24	zyyz	2.31	1.96	1.95	1.95	2.31	1.93	1.93	3.10	1.92	1.96	1.91	2.29	zyxy
2.23	1.92	1.97	2.00	1.91	2.13	1.96	2.23	1.90	1.94	1.91	2.23	zyxy	2.31	1.95	1.96	1.95	1.93	2.31	1.96	2.29	1.91	1.93	1.92	3.10	zyxy
1.91	1.99	2.23	2.23	2.13	1.90	1.89	2.23	1.96	1.93	2.13	1.96	yzzz	1.95	1.96	2.31	2.29	1.91	1.96	1.92	3.10	1.93	1.93	2.31	1.95	zyxy
2.23	1.95	1.94	2.03	2.13	1.89	1.92	2.23	1.92	2.13	1.87	2.14	yzyz	2.31	1.96	1.95	1.95	2.31	1.93	1.93	3.10	1.92	1.96	1.91	2.29	zyxy
2.00	1.91	2.23	2.16	1.87	2.13	1.91	2.23	1.94	2.13	1.89	2.02	yzyx	1.96	1.95	2.31	3.10	1.92	1.93	1.91	2.29	1.96	2.31	1.93	1.95	zyxy
1.97	2.23	1.92	2.18	2.13	1.88	2.13	2.05	1.88	1.88	2.13	2.19	yyyz	1.96	2.31	1.95	3.10	1.93	1.92	2.31	1.95	1.93	1.91	1.96	2.29	zyxy
1.96	1.94	2.23	2.19	2.13	1.89	1.88	2.23	1.98	2.13	1.90	2.00	xzyz	1.96	1.95	2.31	3.10	1.92	1.93	1.91	2.29	1.96	2.31	1.93	1.95	zyxy
1.92	2.01	2.23	2.27	2.13	1.90	2.13	2.24	1.89	1.90	2.13	2.00	xyyz	1.95	1.96	2.31	2.29	1.91	1.96	1.92	3.10	1.93	1.93	2.31	1.95	zyxy

Table S4: Average bond lengths (Å) of the 12 Mn coordination axes before (left) and after (right) full optimization. Values above 2.13Å are highlighted in red, as these indicate a JT axis. The "O–H" bond length is given between the water O attached to the apical Mn and the central proton, where a large value indicates that OH^- is bound to the apical Mn. Values above 1.15Å are highlighted in blue, which indicates a structure with "O" label. The labels on the right are not necessarily corresponding directly to the shown bond lengths, but give the symmetry-equivalent label from Figure S3 or S4 (see also Table S2).

— After pre-optimization —														— After full optimization —													
Mn atom A			Mn atom B			Mn atom C			Mn atom D				Label	Mn atom A			Mn atom B			Mn atom C			Mn atom D				Label
z	*x*	*y*	*z*	*x*	*y*	*z*	*x*	*y*	*z*	*x*	*y*	O–H		*z*	*x*	*y*	*z*	*x*	*y*	*z*	*x*	*y*	*z*	*x*	*y*	O–H	
— Mn4444 —																											
1.88	1.90	1.92	1.96	1.88	1.89	1.88	1.95	1.87	1.88	1.87	1.95	1.53	O4444	1.89	1.91	1.93	1.97	1.88	1.89	1.89	1.96	1.88	1.89	1.88	1.96	1.55	O4444
1.91	1.89	1.91	1.94	1.89	1.90	1.89	1.95	1.88	1.88	1.88	1.95	1.05	H4444	1.92	1.90	1.91	1.94	1.89	1.91	1.89	1.96	1.89	1.89	1.89	1.96	1.06	H4444
— Mn3444 —																											
1.90	2.23	1.99	1.96	1.89	1.87	1.88	1.94	1.89	1.87	1.90	1.95	1.50	Oy444	1.90	2.02	2.16	1.97	1.88	1.90	1.88	1.95	1.91	1.89	1.90	1.95	1.52	Oy444
2.23	1.92	1.93	1.98	1.89	1.90	1.92	1.97	1.87	1.91	1.87	1.97	1.51	Oz444	1.89	1.92	1.93	2.26	1.92	1.93	1.91	1.94	1.89	1.91	1.89	1.94	1.61	O4z44
1.88	1.91	1.92	2.23	1.92	1.92	1.90	1.94	1.88	1.90	1.88	1.94	1.61	O4z44	1.89	1.92	1.93	2.26	1.92	1.93	1.91	1.94	1.89	1.91	1.89	1.94	1.61	O4z44
1.88	1.90	1.94	2.05	1.88	2.23	1.90	1.95	1.87	1.88	1.88	1.96	1.56	O4y44	1.89	1.92	1.93	2.25	1.92	1.93	1.91	1.94	1.89	1.91	1.89	1.94	1.62	O4z44
1.90	1.90	1.91	1.96	1.87	1.89	2.23	2.06	1.87	1.88	1.89	1.95	1.57	O44z4	1.89	1.91	1.93	1.95	1.90	1.90	1.93	2.25	1.92	1.89	1.90	1.94	1.50	O44x4
1.88	1.90	1.94	1.95	1.89	1.88	1.88	2.06	2.23	1.89	1.87	1.96	1.47	O44y4	1.90	1.91	1.93	1.96	1.90	1.90	1.93	2.25	1.92	1.89	1.90	1.94	1.51	O44x4
1.89	1.90	1.92	1.95	1.89	1.89	1.93	2.23	1.91	1.89	1.89	1.94	1.49	O44x4	1.90	1.91	1.93	1.96	1.90	1.90	1.93	2.25	1.92	1.89	1.90	1.94	1.51	O44x4
1.91	1.90	1.91	2.23	1.92	1.92	1.90	1.95	1.88	1.90	1.88	1.95	1.00	H4z44	1.90	1.92	1.92	1.96	1.89	1.91	1.90	1.94	1.90	1.93	1.92	2.24	1.51	O44x4
1.92	1.89	1.92	1.96	1.91	2.23	1.89	1.95	1.87	1.88	1.88	1.97	1.00	H4y44	1.90	1.92	1.92	1.96	1.89	1.91	1.90	1.94	1.90	1.93	1.92	2.24	1.51	O44x4
2.23	1.93	1.94	1.93	1.89	1.90	1.91	1.95	1.87	1.90	1.87	1.95	1.02	Hz444	2.19	1.94	1.95	1.94	1.90	1.91	1.91	1.96	1.88	1.91	1.88	1.96	1.03	Hz444
1.94	2.23	1.95	1.94	1.91	1.88	1.89	1.94	1.89	1.87	1.90	1.95	1.06	Hy444	1.94	2.15	2.00	1.94	1.91	1.90	1.89	1.95	1.90	1.88	1.91	1.95	1.08	Hy444
1.93	1.90	1.90	1.94	1.88	1.90	2.23	2.06	1.87	1.88	1.90	1.94	1.06	H44z4	1.92	1.90	1.92	1.93	1.91	1.90	1.93	2.26	1.92	1.89	1.90	1.94	1.12	H44x4
1.91	1.90	1.93	1.93	1.90	1.89	1.88	2.03	2.23	1.89	1.87	1.96	1.06	H44y4	1.92	1.90	1.92	1.93	1.91	1.90	1.93	2.26	1.92	1.89	1.90	1.94	1.12	H44x4
1.92	1.89	1.91	1.92	1.90	1.90	1.92	2.23	1.92	1.89	1.90	1.94	1.09	H44x4	1.93	1.90	1.92	1.93	1.91	1.91	1.93	2.26	1.92	1.89	1.91	1.94	1.11	H44x4
— Mn3344 —																											
1.91	1.97	2.23	1.97	1.86	1.91	2.23	2.08	1.89	1.88	1.90	1.94	1.57	Oy4z4	1.91	1.97	2.19	1.96	1.89	1.91	1.91	2.26	1.95	1.90	1.91	1.94	1.50	Oy4x4
1.89	1.97	2.23	1.96	1.88	1.90	1.87	2.05	2.23	1.89	1.88	1.95	1.44	Oy4y4	1.91	1.97	2.19	1.96	1.89	1.91	1.91	2.26	1.95	1.90	1.91	1.94	1.50	Oy4x4
1.91	1.95	2.23	1.95	1.87	1.91	1.90	2.23	1.95	1.89	1.90	1.93	1.47	Oy4x4	1.92	1.97	2.20	1.96	1.89	1.92	1.91	2.27	1.96	1.90	1.91	1.94	1.50	Oy4x4
1.93	2.23	1.95	1.97	1.89	1.88	2.23	2.03	1.88	1.87	1.91	1.95	1.54	Ox4z4	1.91	1.97	2.19	1.96	1.89	1.91	1.91	2.26	1.95	1.90	1.91	1.94	1.50	Oy4x4
1.89	2.23	2.03	1.96	1.90	1.87	1.88	2.04	2.23	1.88	1.89	1.96	1.49	Ox4y4	1.91	1.97	2.19	1.96	1.89	1.91	1.91	2.26	1.95	1.90	1.91	1.94	1.51	Oy4x4
1.90	2.23	1.98	1.96	1.90	1.88	1.92	2.23	1.92	1.87	1.91	1.94	1.50	Ox4x4	1.91	1.97	2.19	1.96	1.89	1.91	1.91	2.26	1.95	1.90	1.91	1.94	1.50	Oy4x4
1.95	2.23	1.96	1.97	1.92	2.23	1.90	1.95	1.88	1.87	1.91	1.98	1.00	Hxy44	1.91	2.17	2.00	1.97	1.90	1.90	1.90	1.94	1.91	1.90	1.96	2.25	1.52	Oy4x4
2.23	1.94	1.96	2.23	1.91	1.92	1.91	1.94	1.87	1.91	1.87	1.94	1.55	Ozz44	1.90	2.01	2.18	2.28	1.91	1.95	1.90	1.95	1.91	1.91	1.90	1.94	1.61	Oxz44
1.90	2.23	1.98	2.23	1.95	1.90	1.90	1.94	1.89	1.88	1.90	1.94	1.60	Oxz44	1.90	2.02	2.16	2.28	1.91	1.94	1.89	1.94	1.91	1.90	1.90	1.94	1.62	Oxz44
1.88	2.23	2.03	2.10	1.88	2.23	1.90	1.94	1.89	1.87	1.91	1.96	1.59	Oxy44	1.90	2.01	2.16	2.28	1.91	1.94	1.89	1.94	1.91	1.90	1.90	1.94	1.62	Oxz44
1.91	1.94	2.23	2.07	1.87	2.23	1.89	1.95	1.89	1.88	1.89	1.95	1.51	Oxx44	1.90	2.01	2.17	2.28	1.91	1.95	1.89	1.94	1.91	1.90	1.90	1.94	1.62	Oxz44
1.95	2.23	1.94	2.23	1.96	1.89	1.90	1.94	1.89	1.88	1.91	1.95	1.00	Hxz44	1.90	2.01	2.18	2.28	1.91	1.95	1.90	1.95	1.91	1.91	1.90	1.94	1.61	Oxz44
1.97	1.91	2.23	1.98	1.89	2.23	1.88	1.96	1.89	1.88	1.89	1.96	1.00	Hxx44	1.90	2.01	2.18	2.28	1.91	1.95	1.90	1.95	1.91	1.91	1.90	1.94	1.61	Oxz44
2.23	1.91	1.99	2.04	1.88	2.23	1.92	1.96	1.86	1.90	1.88	1.96	1.52	Ozy44	1.90	1.91	1.94	2.25	1.92	1.94	1.94	2.25	1.92	1.92	1.91	1.93	1.58	O4zx4
2.23	1.95	1.92	1.98	1.88	1.90	2.23	2.05	1.86	1.90	1.88	1.96	1.51	Oz4z4	1.90	1.91	1.94	2.25	1.92	1.94	1.94	2.25	1.92	1.92	1.91	1.93	1.58	O4zx4
2.23	1.91	1.99	1.96	1.90	1.89	1.90	2.11	2.23	1.92	1.87	1.96	1.51	Oz4y4	1.90	1.91	1.94	2.25	1.92	1.94	1.94	2.25	1.92	1.92	1.91	1.93	1.58	O4zx4
2.23	1.92	1.97	1.95	1.89	1.89	1.95	2.23	1.90	1.91	1.88	1.94	1.50	Oz4x4	1.90	1.91	1.94	2.25	1.92	1.94	1.94	2.25	1.92	1.92	1.91	1.93	1.58	O4zx4
1.90	1.92	1.92	2.23	1.89	1.95	2.23	2.00	1.88	1.90	1.89	1.94	1.61	O4zz4	1.89	1.91	1.94	2.24	1.92	1.94	1.93	2.25	1.92	1.91	1.91	1.93	1.58	O4zx4
1.87	1.92	1.94	2.23	1.93	1.91	1.90	2.00	2.23	1.91	1.87	1.95	1.57	O4zy4	1.89	1.91	1.94	2.24	1.92	1.94	1.93	2.25	1.92	1.91	1.91	1.93	1.58	O4zx4
1.89	1.90	1.93	2.23	1.91	1.94	1.93	2.23	1.92	1.91	1.90	1.93	1.56	O4zx4	1.89	1.91	1.94	2.25	1.92	1.94	1.93	2.25	1.92	1.91	1.91	1.93	1.59	O4zx4
1.91	1.90	1.93	2.04	1.87	2.23	2.23	2.05	1.87	1.88	1.90	1.96	1.59	O4yz4	1.90	1.91	1.94	2.25	1.92	1.94	1.94	2.25	1.92	1.92	1.91	1.93	1.58	O4zx4
1.88	1.90	1.96	2.03	1.89	2.23	1.88	2.04	2.23	1.88	1.88	1.97	1.50	O4yy4	1.89	1.91	1.94	2.24	1.92	1.94	1.93	2.25	1.92	1.91	1.91	1.93	1.58	O4zx4
1.89	1.89	1.94	2.00	1.89	2.23	1.94	2.23	1.90	1.88	1.90	1.95	1.51	O4yx4	1.89	1.91	1.94	2.24	1.92	1.94	1.93	2.25	1.92	1.91	1.91	1.93	1.58	O4zx4
1.90	1.93	1.89	1.97	2.23	1.89	2.23	1.97	1.88	1.90	1.89	1.95	1.74	O4xz4	1.89	1.91	1.94	2.24	1.92	1.94	1.93	2.25	1.92	1.91	1.91	1.93	1.58	O4zx4
1.88	1.93	1.93	2.04	2.23	1.88	1.87	2.05	2.23	1.90	1.87	1.96	1.51	O4xy4	1.89	1.91	1.94	2.24	1.92	1.94	1.93	2.25	1.92	1.91	1.91	1.93	1.58	O4zx4
1.89	1.92	1.92	2.00	2.23	1.90	1.90	2.23	1.93	1.90	1.88	1.94	1.53	O4xx4	1.90	1.91	1.94	2.25	1.92	1.94	1.94	2.25	1.92	1.92	1.91	1.93	1.58	O4zx4
1.94	1.90	1.91	2.23	1.89	1.96	2.23	2.00	1.89	1.90	1.90	1.95	1.00	H4zz4	1.90	1.91	1.94	2.25	1.92	1.94	1.94	2.25	1.92	1.92	1.91	1.93	1.58	O4zx4
1.91	1.91	1.94	2.23	1.93	1.92	1.91	2.00	2.23	1.92	1.88	1.97	1.00	H4zy4	1.90	1.91	1.94	2.25	1.92	1.94	1.94	2.25	1.92	1.92	1.91	1.93	1.58	O4zx4
1.92	1.90	1.92	2.23	1.92	1.94	1.92	2.23	1.92	1.91	1.90	1.93	1.00	H4zx4	1.90	1.91	1.94	2.25	1.92	1.94	1.94	2.25	1.92	1.92	1.91	1.93	1.58	O4zx4
1.95	1.89	1.92	1.98	1.90	2.23	2.23	2.05	1.87	1.87	1.90	1.96	1.00	H4yz4	1.90	1.91	1.94	2.25	1.92	1.94	1.94	2.25	1.92	1.92	1.91	1.93	1.58	O4zx4
1.91	1.89	1.95	1.98	1.91	2.23	1.88	2.05	2.23	1.88	1.88	1.97	1.11	H4yy4	1.89	1.91	1.94	2.24	1.92	1.94	1.93	2.25	1.92	1.91	1.91	1.93	1.58	O4zx4
1.93	1.88	1.93	1.94	1.93	2.23	1.93	2.23	1.91	1.88	1.90	1.95	1.00	H4yx4	1.90	1.91	1.94	2.25	1.92	1.94	1.94	2.25	1.92	1.92	1.91	1.93	1.58	O4zx4
1.91	1.92	1.92	1.99	2.23	1.90	1.87	2.06	2.23	1.90	1.87	1.96	1.11	H4xy4	1.90	1.91	1.94	2.25	1.92	1.94	1.94	2.25	1.92	1.92	1.91	1.93	1.58	O4zx4
1.91	1.93	1.89	1.92	2.23	1.90	1.87	2.23	1.88	1.89	1.90	1.93	1.05	H4xx4	1.89	1.91	1.94	2.24	1.92	1.94	1.93	2.25	1.92	1.91	1.91	1.93	1.58	O4zx4

Table S5: Average bond lengths (Å) of the 12 Mn coordination axes before (left) and after (right) full optimization. Values above 2.13Å are highlighted in red, as these indicate a JT axis. The “O–H” bond length is given between the water O attached to the apical Mn and the central proton, where a large value indicates that OH^- is bound to the apical Mn. Values above 1.15Å are highlighted in blue, which indicates a structure with “O” label. The labels on the right are not necessarily corresponding directly to the shown bond lengths, but give the symmetry-equivalent label from Figure S3 or S4 (see also Table S2.

— After pre-optimization —														— After full optimization —													
Mn atom A			Mn atom B			Mn atom C			Mn atom D				Label	Mn atom A			Mn atom B			Mn atom C			Mn atom D				Label
z	*x*	*y*	*z*	*x*	*y*	*z*	*x*	*y*	*z*	*x*	*y*	O–H		*z*	*x*	*y*	*z*	*x*	*y*	*z*	*x*	*y*	*z*	*x*	*y*	O–H	
— Mn3344 (cont.d) —																											
1.92	1.90	1.91	1.98	1.88	1.89	2.23	2.05	1.88	2.23	1.88	2.04	1.62	O44zz	1.91	1.91	1.93	1.95	1.91	1.92	1.94	2.24	1.92	1.94	1.92	2.20	1.50	O44xy
1.91	1.92	1.91	1.96	1.88	1.91	2.23	2.01	1.89	1.92	1.92	2.23	1.55	O44zy	1.91	1.91	1.93	1.94	1.91	1.92	1.94	2.25	1.92	1.94	1.92	2.23	1.50	O44xy
1.88	1.93	1.91	1.97	1.87	1.89	2.23	1.97	1.85	1.89	2.23	1.95	1.61	O44zx	1.91	1.91	1.93	1.94	1.91	1.92	1.94	2.25	1.92	1.94	1.92	2.23	1.50	O44xy
1.87	1.94	1.95	1.96	1.89	1.89	1.88	2.06	2.23	1.88	2.23	2.05	1.56	O44yx	1.91	1.92	1.93	1.95	1.91	1.92	1.94	2.25	1.92	1.94	1.92	2.23	1.50	O44xy
1.90	1.91	1.92	1.94	1.90	1.91	1.93	2.23	1.91	1.93	1.92	2.23	1.49	O44xy	1.91	1.91	1.93	1.94	1.91	1.92	1.94	2.25	1.92	1.94	1.92	2.23	1.50	O44xy
1.89	1.92	1.93	1.95	1.90	1.90	1.89	1.99	2.23	1.95	1.89	2.23	1.45	O44xx	1.91	1.91	1.93	1.94	1.91	1.92	1.94	2.25	1.92	1.94	1.92	2.23	1.50	O44xy
1.93	1.92	1.87	1.96	2.23	1.90	2.23	1.97	1.88	1.90	1.89	1.95	1.05	H4xz4	1.91	1.91	1.93	1.94	1.91	1.92	1.94	2.25	1.92	1.94	1.92	2.23	1.50	O44xy
1.96	1.89	1.90	1.94	1.89	1.90	2.23	2.03	1.88	2.23	1.88	2.02	1.00	H44zz	1.91	1.92	1.93	1.95	1.91	1.92	1.94	2.25	1.92	1.94	1.92	2.23	1.50	O44xy
1.94	1.91	1.89	1.93	1.88	1.92	2.23	1.99	1.89	1.91	1.93	2.23	1.10	H44zy	1.91	1.91	1.93	1.94	1.91	1.92	1.94	2.25	1.92	1.94	1.92	2.23	1.50	O44xy
1.93	1.92	1.90	1.93	1.88	1.91	2.23	2.05	1.87	1.88	2.23	2.03	1.07	H44zx	1.91	1.92	1.93	1.95	1.91	1.92	1.94	2.25	1.92	1.94	1.92	2.23	1.50	O44xy
1.90	1.93	1.94	1.93	1.89	1.90	1.88	2.03	2.23	1.89	2.23	2.02	1.05	H44yx	1.91	1.91	1.93	1.94	1.91	1.92	1.94	2.25	1.92	1.94	1.92	2.23	1.50	O44xy
1.93	1.90	1.90	1.90	1.91	1.92	1.93	2.23	1.92	1.93	1.92	2.23	1.00	H44xy	1.91	1.92	1.93	1.95	1.91	1.92	1.94	2.25	1.92	1.94	1.92	2.23	1.50	O44xy
1.92	1.91	1.92	1.92	1.90	1.90	1.90	1.98	2.23	1.94	1.90	2.23	1.09	H44xx	1.91	1.91	1.93	1.94	1.91	1.92	1.94	2.25	1.92	1.94	1.92	2.23	1.50	O44xy
2.23	1.94	1.95	2.23	1.92	1.93	1.92	1.95	1.87	1.92	1.87	1.95	1.15	Hzz44	2.21	1.95	1.94	1.93	1.90	1.93	1.92	1.95	1.89	1.96	1.90	2.26	1.05	Hz4x4
2.23	1.95	1.93	1.93	1.89	1.90	2.23	2.05	1.86	1.90	1.88	1.95	1.03	Hz4z4	2.19	1.93	1.97	1.92	1.91	1.91	1.96	2.27	1.90	1.91	1.89	1.94	1.04	Hz4x4
2.23	1.92	1.97	1.93	1.90	1.90	1.89	2.07	2.23	1.92	1.87	1.96	1.04	Hz4y4	2.19	1.93	1.97	1.92	1.91	1.91	1.95	2.27	1.90	1.91	1.89	1.94	1.04	Hz4x4
2.23	1.92	1.95	1.92	1.90	1.90	1.96	2.23	1.89	1.91	1.88	1.94	1.04	Hz4x4	2.21	1.93	1.96	1.92	1.91	1.92	1.97	2.27	1.90	1.92	1.90	1.95	1.04	Hz4x4
1.93	1.90	2.23	1.95	1.85	1.93	2.23	1.95	1.90	1.90	1.88	1.97	1.05	Hy4z4	1.96	1.94	2.19	1.93	1.90	1.93	1.91	2.27	1.95	1.90	1.91	1.93	1.08	Hy4x4
1.93	1.96	2.23	1.93	1.89	1.91	1.87	2.06	2.23	1.89	1.88	1.95	1.07	Hy4y4	1.96	1.94	2.19	1.93	1.90	1.93	1.90	2.27	1.95	1.90	1.91	1.93	1.08	Hy4x4
1.95	1.93	2.23	1.92	1.88	1.92	1.90	2.23	1.95	1.89	1.90	1.93	1.07	Hy4x4	1.96	1.94	2.20	1.93	1.90	1.93	1.91	2.27	1.96	1.90	1.91	1.94	1.11	Hy4x4
1.92	2.23	2.00	1.93	1.91	1.88	1.88	2.03	2.23	1.88	1.89	1.96	1.07	Hx4y4	1.96	1.94	2.19	1.93	1.90	1.93	1.90	2.27	1.95	1.90	1.91	1.94	1.08	Hy4x4
2.23	1.91	1.97	1.97	1.90	2.23	1.91	1.95	1.86	1.89	1.87	1.97	1.04	Hzy44	1.95	1.96	2.18	1.93	1.89	1.94	1.89	1.95	1.93	1.92	1.93	2.25	1.12	Hx4x4
1.99	2.23	1.92	1.94	1.90	1.89	2.23	2.02	1.88	1.87	1.91	1.95	1.06	Hx4z4	1.94	2.15	1.99	1.93	1.92	1.90	1.92	2.25	1.93	1.89	1.92	1.94	1.09	Hx4x4
1.95	2.23	1.95	1.93	1.91	1.88	1.92	2.23	1.92	1.88	1.91	1.94	1.07	Hx4x4	1.94	2.15	2.00	1.94	1.92	1.90	1.92	2.25	1.94	1.89	1.93	1.95	1.13	Hx4x4
— Mn3334 —																											
2.23	1.93	1.98	2.23	1.91	1.94	1.95	2.23	1.90	1.92	1.89	1.94	1.54	Ozzx4	1.91	1.97	2.23	2.28	1.91	1.97	1.91	2.27	1.97	1.92	1.92	1.94	1.59	Oyzx4
1.90	1.97	2.23	2.23	1.90	1.96	1.90	2.23	1.96	1.90	1.90	1.93	1.62	Oyzx4	1.91	1.97	2.24	2.28	1.91	1.97	1.91	2.27	1.97	1.92	1.92	1.94	1.59	Oyzx4
1.90	2.23	1.99	2.23	1.95	1.90	1.92	2.23	1.93	1.89	1.92	1.94	1.66	Oxzx4	1.91	1.97	2.23	2.28	1.91	1.97	1.91	2.27	1.97	1.92	1.92	1.93	1.59	Oyzx4
2.23	1.92	1.97	2.23	1.90	1.97	1.96	2.23	1.89	1.92	1.89	1.94	1.00	Hzzx4	1.91	1.97	2.23	2.28	1.91	1.97	1.91	2.27	1.97	1.92	1.92	1.93	1.59	Oyzx4
1.96	1.93	2.23	2.23	1.90	1.98	1.90	2.23	1.95	1.91	1.90	1.93	1.00	Hyzx4	1.91	1.97	2.23	2.28	1.91	1.97	1.91	2.27	1.97	1.92	1.92	1.93	1.59	Oyzx4
1.95	2.23	1.96	2.23	1.97	1.90	1.91	2.23	1.94	1.89	1.91	1.94	1.00	Hxzx4	1.91	1.97	2.23	2.28	1.91	1.97	1.91	2.27	1.97	1.92	1.92	1.93	1.59	Oyzx4
1.91	1.97	2.23	1.96	1.89	1.92	1.91	2.23	1.95	1.93	1.92	2.23	1.61	Oy4xy	1.93	1.97	2.21	1.97	1.90	1.94	1.92	2.29	1.95	1.94	1.93	2.25	1.52	Oy4xy
1.97	1.93	2.23	1.91	1.90	1.93	1.90	2.23	1.95	1.93	1.91	2.23	1.00	Hy4xy	1.93	1.97	2.21	1.97	1.90	1.94	1.92	2.29	1.95	1.94	1.93	2.25	1.52	Oy4xy
1.89	1.92	1.93	2.23	1.92	1.93	1.93	2.23	1.91	1.93	1.91	2.23	1.60	O4zxy	1.91	1.92	1.94	2.27	1.93	1.94	1.94	2.25	1.92	1.94	1.93	2.24	1.58	O4zxy
1.93	1.91	1.91	2.23	1.92	1.93	1.93	2.23	1.92	1.93	1.92	2.23	1.00	H4zxy	1.91	1.92	1.94	2.27	1.93	1.94	1.94	2.25	1.92	1.95	1.93	2.24	1.58	O4zxy
2.23	1.94	1.95	1.95	1.90	1.91	1.97	2.23	1.90	1.96	1.90	2.23	1.52	Oz4xy	2.25	1.94	1.95	1.92	1.92	1.93	1.98	2.26	1.90	1.97	1.91	2.26	1.06	Hz4xy
2.23	1.93	1.94	1.91	1.91	1.92	1.98	2.23	1.89	1.96	1.89	2.23	1.00	Hz4xy	2.25	1.94	1.95	1.92	1.92	1.93	1.98	2.26	1.90	1.97	1.91	2.26	1.06	Hz4xy
— Mn3333 —																											
2.23	1.95	1.96	2.23	1.92	1.93	1.96	2.23	1.90	1.96	1.90	2.23	1.55	Ozzxy	1.91	2.25	1.99	2.91	1.94	1.91	1.95	2.25	1.93	1.92	1.95	2.29	1.80	Oyzxy
1.91	1.97	2.23	2.23	1.90	1.95	1.90	2.23	1.95	1.92	1.92	2.23	1.63	Oyzxy	1.91	2.25	1.99	2.91	1.94	1.91	1.96	2.25	1.93	1.92	1.95	2.29	1.80	Oyzxy
2.23	1.94	1.95	2.23	1.92	1.93	1.96	2.23	1.89	1.96	1.90	2.23	1.00	Hzzxy	1.91	2.25	1.99	2.91	1.94	1.91	1.96	2.25	1.93	1.92	1.95	2.29	1.80	Oyzxy
1.97	1.95	2.23	2.23	1.90	1.97	1.91	2.23	1.96	1.93	1.93	2.23	1.00	Hyzxy	1.91	2.25	1.99	2.91	1.94	1.91	1.96	2.25	1.93	1.92	1.95	2.29	1.80	Oyzxy

S4 Molecular orbitals

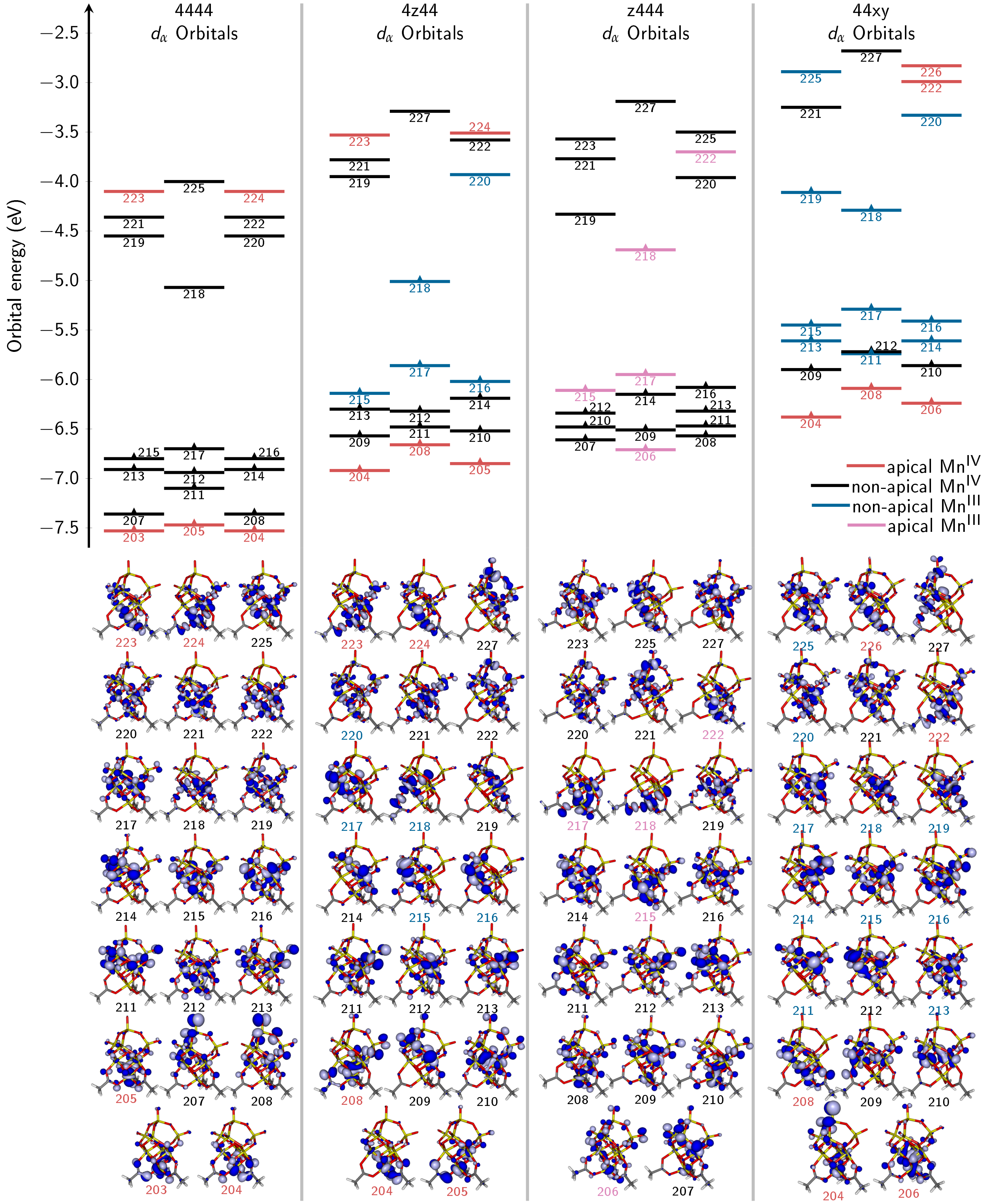

Figure S5: Kohn–Sham orbital energies (top) and Kohn–Sham molecular orbital depictions of the 20 Mn d_α orbitals (bottom). The orbital energies are computed at the BP86/mixed basis level of theory (Gaussian 16) described in the main text. The orbitals are shown for the four minima 4444, 4z44, z444, and 44xy. Colors indicate whether some orbital is mostly localized on certain Mn atoms (see legend on the right).

S5 Population analysis

Table S6: Atomic charges of selected atoms from Mulliken population analysis for all twelve JT arrangements of the precatalyst. Mn_a is the apical Mn, O_a the apical O, O_{ac1} an acetate O connected to Mn_a, O_{ac2} an acetate O connected to Mn, O_{v1} a vanadate O connected to Mn, and O_{v2} a vandate O bridging two V atoms.

Label	Mn_a	Mn			O_{cub}			O_a	O_{ac1}			O_{ac2}			O_{v1}						O_{v2}		
4444	0.54	0.66	0.67	0.66	-0.33	-0.33	-0.33	-0.53	-0.30	-0.30	-0.30	-0.27	-0.27	-0.27	-0.56	-0.55	-0.56	-0.56	-0.56	-0.56	-0.74	-0.74	-0.74
z444	0.78	0.63	0.64	0.63	-0.38	-0.41	-0.41	-0.55	-0.38	-0.41	-0.38	-0.29	-0.30	-0.29	-0.57	-0.57	-0.57	-0.57	-0.57	-0.58	-0.75	-0.76	-0.76
4z44	0.52	0.66	0.82	0.66	-0.37	-0.41	-0.40	-0.58	-0.31	-0.33	-0.31	-0.30	-0.37	-0.29	-0.56	-0.57	-0.58	-0.65	-0.65	-0.59	-0.74	-0.75	-0.75
yz44	0.74	0.64	0.79	0.64	-0.44	-0.49	-0.48	-0.60	-0.40	-0.42	-0.42	-0.31	-0.40	-0.32	-0.58	-0.59	-0.60	-0.66	-0.66	-0.59	-0.76	-0.76	-0.76
zz44	0.74	0.64	0.80	0.64	-0.42	-0.50	-0.49	-0.59	-0.40	-0.45	-0.40	-0.32	-0.42	-0.31	-0.58	-0.58	-0.60	-0.66	-0.66	-0.60	-0.76	-0.76	-0.76
44xy	0.51	0.81	0.67	0.81	-0.49	-0.43	-0.43	-0.67	-0.34	-0.32	-0.34	-0.40	-0.32	-0.40	-0.67	-0.67	-0.65	-0.59	-0.59	-0.65	-0.76	-0.75	-0.75
4zxy	0.50	0.81	0.81	0.81	-0.50	-0.50	-0.50	-0.82	-0.35	-0.35	-0.35	-0.42	-0.42	-0.42	-0.68	-0.68	-0.68	-0.68	-0.68	-0.68	-0.74	-0.74	-0.74
4yxy	0.51	0.81	0.81	0.81	-0.52	-0.52	-0.50	-0.75	-0.36	-0.34	-0.36	-0.43	-0.39	-0.43	-0.68	-0.68	-0.68	-0.67	-0.69	-0.69	-0.77	-0.75	-0.76
z4xy	0.72	0.79	0.66	0.79	-0.57	-0.51	-0.51	-0.70	-0.43	-0.45	-0.43	-0.43	-0.35	-0.43	-0.68	-0.68	-0.66	-0.60	-0.61	-0.66	-0.77	-0.76	-0.76
y4xy	0.72	0.79	0.65	0.79	-0.58	-0.51	-0.51	-0.69	-0.46	-0.41	-0.43	-0.44	-0.34	-0.43	-0.68	-0.68	-0.66	-0.61	-0.61	-0.66	-0.77	-0.75	-0.76
zzxy	0.71	0.80	0.80	0.81	-0.60	-0.59	-0.60	-0.83	-0.48	-0.43	-0.43	-0.46	-0.44	-0.45	-0.69	-0.69	-0.69	-0.69	-0.69	-0.69	-0.74	-0.74	-0.74
zyxy	0.73	0.79	0.77	0.84	-0.62	-0.64	-0.65	-0.79	-0.45	-0.43	-0.42	-0.42	-0.54	-0.44	-0.69	-0.70	-0.68	-0.67	-0.68	-0.69	-0.77	-0.76	-0.78

Table S7: Atomic spin populations of selected atoms from Mulliken population analysis for all twelve JT arrangements of the precatalyst.

Label	Mn_a	Mn			O_{cub}			O_a	O_{ac1}			O_{ac2}			O_{v1}						O_{v2}		
4444	2.89	2.80	2.80	2.80	-0.02	-0.02	-0.02	-0.07	0.01	0.01	0.01	0.02	0.02	0.02	0.07	0.07	0.07	0.07	0.07	0.07	0.00	0.00	0.00
z444	3.84	2.86	2.85	2.86	0.12	-0.04	-0.04	-0.06	0.01	0.05	0.01	0.00	0.00	0.00	0.04	0.04	0.04	0.05	0.04	0.04	0.00	0.00	0.00
4z44	2.91	2.87	3.75	2.87	-0.03	-0.01	-0.01	0.03	0.00	0.00	0.00	0.01	0.05	0.01	0.04	0.04	0.05	0.01	0.01	0.05	0.00	0.00	0.00
yz44	3.84	2.91	3.81	2.91	-0.05	-0.02	0.09	0.03	0.00	-0.01	0.04	0.00	0.04	0.00	0.02	0.01	0.02	0.00	0.00	0.02	0.00	0.00	0.00
zz44	3.84	2.93	3.76	2.93	0.11	-0.05	-0.05	0.03	0.00	0.04	0.00	0.00	0.04	0.00	0.01	0.01	0.01	0.00	0.00	0.01	0.00	0.00	0.00
44xy	2.93	3.77	2.88	3.77	-0.02	-0.01	-0.01	0.19	-0.01	-0.01	-0.01	0.04	0.00	0.04	0.01	0.01	0.00	0.01	0.01	0.00	0.00	0.00	0.00
4zxy	2.95	3.73	3.73	3.74	-0.02	-0.02	-0.02	0.25	-0.01	-0.01	-0.01	0.03	0.03	0.03	0.00	0.00	0.00	0.00	0.00	0.00	0.00	0.00	0.00
4yxy	2.93	3.79	3.75	3.78	-0.01	-0.02	0.11	0.17	-0.02	-0.01	-0.02	0.03	-0.01	0.03	0.00	-0.01	0.00	-0.01	0.01	0.01	0.00	0.00	0.00
z4xy	3.86	3.81	2.92	3.82	0.07	-0.03	-0.03	0.18	-0.01	0.03	-0.01	0.03	-0.02	0.03	-0.01	-0.01	-0.01	0.00	-0.01	-0.01	0.00	0.00	0.00
y4xy	3.85	3.82	2.91	3.80	-0.03	0.08	-0.05	0.19	0.03	0.00	-0.02	0.03	-0.01	0.03	-0.01	0.00	-0.01	-0.01	-0.01	-0.01	0.00	0.00	0.00
zzxy	3.88	3.78	3.78	3.77	-0.04	0.07	-0.04	0.18	0.02	-0.01	-0.02	0.02	0.02	0.02	-0.01	-0.01	-0.01	-0.01	-0.01	-0.01	0.00	0.00	0.00
zyxy	3.90	3.83	3.78	3.80	-0.04	0.07	0.08	0.19	0.02	-0.02	-0.02	-0.02	0.01	0.02	-0.01	0.02	-0.02	-0.01	-0.02	-0.01	0.00	0.00	0.00

S6 Predicted energies

Table S8: Overview over the energies of different JT arrangements of the precatalyst as predicted by the four rules (apical JT axis, two- and three-axes crossings, and vanadate JT axes) discussed in the main text.

Structure	Apical JT axis (kcal/mol)	Two-axis crossing (kcal/mol)	Three-axis crossing (kcal/mol)	Vanadate JT axis (kcal/mol)	E_{predict} (kcal/mol)	$E_{\text{pred., rel}}$ (kcal/mol)	E_{DFT} (kcal/mol)
				— Mn3444 —			
4z44	0	0	0	0	0	0	0.0
z444	+10	0	0	0	10	10	9.7
4x44	0	0	0	+12	12	12	
				— Mn3344 —			
44xy	0	+6	0	0	6	0	0.0
yz44	+10	0	0	0	10	4	3.9
zz44	+10	0	0	0	10	4	4.4
44xx	0	0	0	+12	12	6	
44xz	0	0	0	+12	12	6	
yy44	+10	0	0	+12	22	16	
zy44	+10	0	0	+12	22	16	
44yz	0	0	0	+24	24	18	
44zz	0	0	0	+24	24	18	
xy44	+10	+6	0	+12	28	22	
44yx	0	+6	0	+24	30	24	
				— Mn3334 —			
z4xy	+10	+6	0	0	16	0	0.0
y4xy	+10	+6	0	0	16	0	0.05
4yxy	0	+6	0	+12	18	2	2.2
4zxy	0	0	+19	0	19	3	3.2
z4xz	+10	0	0	+12	22	6	
y4xz	+10	0	0	+12	22	6	
y4xx	+10	0	0	+12	22	6	
x4xx	+10	0	0	+12	22	6	
4zzz	0	0	0	+24	24	8	
4zzx	0	0	0	+24	24	8	
z4xx	+10	+6	0	+12	28	12	
x4xz	+10	+6	0	+12	28	12	
4zyx	0	+6	0	+24	30	14	
z4zz	+10	0	0	+24	34	18	
y4yz	+10	0	0	+24	34	18	
4yzx	0	0	0	+36	36	20	
x4zz	+10	+6	0	+24	40	24	
y4yx	+10	+6	0	+24	40	24	
z4yz	+10	+6	0	+24	40	24	
x4yz	+10	+6	0	+24	40	24	
4yyx	0	+6	0	+36	42	26	
z4yx	+10	0	+19	+24	53	37	
				— Mn3333 —			
zyxy	+10	+6	0	+12	28	0	0.0
yyxy	+10	+6	0	+12	28	0	
zzxy	+10	0	+19	0	29	1	5.1[a]
xyxy	+10	+12	0	+12	34	6	
zzzz	+10	+0	0	+24	34	6	
yzyz	+10	0	0	+24	34	6	
xzzz	+10	+6	0	+24	40	12	
yzyx	+10	+6	0	+24	40	12	
zzyz	+10	+6	0	+24	40	12	
xzyz	+10	+6	0	+24	40	12	
yyyz	+10	+6	0	+36	52	24	
zyzx	+10	+6	0	+36	52	24	
zzyx	+10	0	+19	+24	53	25	
zyyz	+10	+12	0	+36	58	30	
xyyz	+10	0	+19	+36	65	37	

[a] Optimized structure dissociates one Mn–O bond.

Table S9: Overview over the energies of different JT arrangements of the catalyst as predicted by the adjusted four rules (apical JT axis, two- and three-axes crossings, and vanadate JT axes) and further influences discussed in the main text.

Structure	Apical JT axis (kcal/mol)	Two-axis crossing (kcal/mol)	Three-axis crossing (kcal/mol)	Vanadate JT axis (kcal/mol)	E_{predict} (kcal/mol)	$E_{\text{pred., rel}}$ (kcal/mol)	$E_{\text{DFT,O}}$ (kcal/mol)	$E_{\text{DFT,H}}$ (kcal/mol)
				— Mn4444 —				
4444	0	0	0	0	0	0	0.00	0.24
				— Mn3444 —				
44x4	0	0	0	0	0	0	0.00	0.37
4z44	0	0	0	0	0	0	0.51	—[a]
z444	+7.5	0	0	0	7.5	7.5	—[a]	7.70
y444	+7.5	0	0	0	7.5	7.5	7.46	7.67
4y44	0	0	0	+12	12	12		
44y4	0	0	0	+12	12	12		
44z4	0	0	0	+12	12	12		
				— Mn3344 —				
4zx4	0	+5	0	0	5	0	0.00	—[a]
44xy	0	+5	0	0	5	0	0.22	
xz44	+7.5	0	0	0	7.5	2.5	2.67	—[a]
z4x4	+7.5	0	0	0	7.5	2.5	—[a]	4.21
y4x4	+7.5	0	0	0	7.5	2.5	2.63	2.81
x4x4	+7.5	0	0	0	7.5	2.5		2.00
zz44	+7.5	0	0	0	7.5	2.5	—[a]	—[a]
44zy	0	0	0	+12	12	7		
44xx	0	0	0	+12	12	7		
4zz4	0	0	0	+12	12	7		
4zy4	0	0	0	+12	12	7		
4yx4	0	0	0	+12	12	7		
4xx4	0	0	0	+12	12	7		
zy44	+7.5	0	0	+12	19.5	14.5		
xx44	+7.5	0	0	+12	19.5	14.5		
z4z4	+7.5	0	0	+12	19.5	14.5		
y4y4	+7.5	0	0	+12	19.5	14.5		
x4z4	+7.5	0	0	+12	19.5	14.5		
x4y4	+7.5	0	0	+12	19.5	14.5		
44zz	0	0	0	+24	24	19		
44zx	0	0	0	+24	24	19		
4yz4	0	0	0	+24	24	19		
4yy4	0	0	0	+24	24	19		
4xy4	0	0	0	+24	24	19		
xy44	+7.5	+5	0	+12	24.5	19.5		
z4y4	+7.5	+5	0	+12	24.5	19.5		
y4z4	+7.5	+5	0	+12	24.5	19.5		
44yx	0	+5	0	+24	29	24		
4xz4	0	+5	0	+24	29	24		

[a] Presumably absent due to containing a JT axis towards the hydroxide ligand.

Table S10: (continued) Overview over the energies of different JT arrangements of the catalyst as predicted by the adjusted four rules (apical JT axis, two- and three-axes crossings, and vanadate JT axes) and further influences discussed in the main text.

Structure	Apical JT axis (kcal/mol)	Two-axis crossing (kcal/mol)	Three-axis crossing (kcal/mol)	Vanadate JT axis (kcal/mol)	$E_{predict}$ (kcal/mol)	$E_{pred., rel}$ (kcal/mol)	$E_{DFT,O}$ (kcal/mol)	$E_{DFT,H}$ (kcal/mol)
				— Mn3334 —				
yzx4	+7.5	+5	0	0	12.5	0	0.00	—[a]
y4xy	+7.5	+5	0	0	12.5	0	1.52	
z4xy	+7.5	+5	0	0	12.5	0	—[a]	2.22
zzx4	+7.5	+5	0	0	12.5	0	—[a]	—[a]
xzx4	+7.5	+5	0	0	12.5	0		—[a]
4zxy	0	0	+17	0	17	4.5	4.20	—[a]
4xxy	0	+5	0	+12	17	4.5		
4zxx	0	+5	0	+12	17	4.5		
4zzy	0	+5	0	+12	17	4.5		
x4xx	+7.5	0	0	+12	19.5	7		
x4xz	+7.5	0	0	+12	19.5	7		
x4yy	+7.5	0	0	+12	19.5	7		
x4zy	+7.5	0	0	+12	19.5	7		
xxx4	+7.5	0	0	+12	19.5	7		
xzy4	+7.5	0	0	+12	19.5	7		
xzz4	+7.5	0	0	+12	19.5	7		
yyx4	+7.5	0	0	+12	19.5	7		
yzy4	+7.5	0	0	+12	19.5	7		
yzz4	+7.5	0	0	+12	19.5	7		
z4zy	+7.5	0	0	+12	19.5	7		
zxx4	+7.5	0	0	+12	19.5	7		
zyx4	+7.5	0	0	+12	19.5	7		
zzz4	+7.5	0	0	+12	19.5	7		
4xxx	0	0	0	+24	24	11.5		
4xxz	0	0	0	+24	24	11.5		
4xyy	0	0	0	+24	24	11.5		
4xzy	0	0	0	+24	24	11.5		
4zzx	0	0	0	+24	24	11.5		
4zzz	0	0	0	+24	24	11.5		
xyx4	+7.5	+5	0	+12	24.5	12		
yxx4	+7.5	+5	0	+12	24.5	12		
z4xx	+7.5	+5	0	+12	24.5	12		
zzy4	+7.5	+5	0	+12	24.5	12		
4zyx	0	0	+17	+12	29	16.5		
x4yz	+7.5	0	0	+24	31.5	19		
x4zx	+7.5	0	0	+24	31.5	19		
x4zz	+7.5	0	0	+24	31.5	19		
xxy4	+7.5	0	0	+24	31.5	19		
xxz4	+7.5	0	0	+24	31.5	19		
yyy4	+7.5	0	0	+24	31.5	19		
yyz4	+7.5	0	0	+24	31.5	19		
z4zz	+7.5	0	0	+24	31.5	19		
zxz4	+7.5	0	0	+24	31.5	19		
zyz4	+7.5	0	0	+24	31.5	19		
4xyz	0	0	0	+36	36	23.5		
4xzx	0	0	0	+36	36	23.5		
4xzz	0	0	0	+36	36	23.5		
x4yx	+7.5	+5	0	+24	36.5	24		
xyy4	+7.5	+5	0	+24	36.5	24		
xyz4	+7.5	+5	0	+24	36.5	24		
yxy4	+7.5	+5	0	+24	36.5	24		
z4zx	+7.5	+5	0	+24	36.5	24		
zxy4	+7.5	+5	0	+24	36.5	24		
zyy4	+7.5	+5	0	+24	36.5	24		
4xyx	0	+5	0	+36	41	28.5		
z4yx	+7.5	0	+17	+24	48.5	36		
yxz4	+7.5	0	+17	+24	48.5	36		

[a] Presumably absent due to containing a JT axis towards the hydroxide ligand.

S7 Error estimation for heuristic rules

The MnV WOC is a challenging system where large errors in the computed energies could hypothetically occur. In order to estimate the uncertainty in the computed electronic energies, we performed single point calculations at all optimized minima of the precatalyst. Note that we did not reoptimize the structure and reuse the free energy corrections from the computations presented in the main manuscript, which use BP86 and def2-SVP for C, H and def2-TZVP for Mn, V, O. The single point energies below, computed with BP86/def2-SVP (smal basis), B3LYP/mixed basis, and CAM-B3LYP/mixed basis, were then combined with the free energy corrections. The results are shown in Table S11.

It can be observed in the table that the results from different basis sets and functionals are mostly consistent. The relative free energy of the z444 structure varies by about 1 kcal/mol (between 9.2 and 10.4 kcal/mol). All computations thus agree on the approximate cost of 9–10 kcal/mol of an apical JT axis (rule 1). The relative free energies of the Mn3344 minima is also well reproduced by all computations, with a variation of about 1.5 kcal/mol; the relative ordering is preserved in all cases. Thus, the cost of a JT axis crossing is about 6–7 kcal/mol (rule 2). For Mn3334, the results show slightly more deviations. The small basis set predicts the wrong energetic order between z4xy and y4xy, although the error is only on the order of 0.2 kcal/mol. Notably, the two hybrid functionals (B3LYP and CAM-B3LYP) predict a much higher energy of the 4zxy structure, with a deviation of about 4 kcal/mol. Based on these results, a vanadate JT axis appears to cost about 12–14 kcal/mol (rule 3). A triple JT axis crossing is predicted to cost 19 kcal/mol with BP86, but 22–23 kcal/mol with B3LYP or CAM-B3LYP (rule 4), which is the heuristic rule with the largest uncertainty.

Note that there are no optimized structures that could be used to estimate the cost of a hydroxide JT axis, and hence it is not easily possible to scrutinize the uncertainty of rule 5.

Table S11: Relative free energies of the 12 optimized minima of the precatalyst, based on optimizations and free energy corrections from BP86/large basis and single point electronic energies from the given four levels of theory.

Label	Conf.	ΔG (kcal/mol) BP86/large[a]	ΔG (kcal/mol) BP86/small[a]	ΔG (kcal/mol) B3LYP/large[a]	ΔG (kcal/mol) CAM-B3LYP/large[a]
Mn4444	4444	0.0	0.0	0.0	0.0
Mn3444	4z44	0.0	0.0	0.0	0.0
	z444	9.7	10.4	9.2	10.4
Mn3344	44xy	0.0	0.0	0.0	0.0
	yz44	3.9	4.6	3.0	3.4
	zz44	4.4	4.8	4.0	4.4
Mn3334	z4xy	0.0	0.0	0.0	0.0
	y4xy	0.05	−0.15	0.3	0.1
	4yxy	2.2	3.1	3.5	3.5
	4zxy	3.2	3.5	7.2	7.5
Mn3333	zyxy	0.0	0.0	0.0	0.0
	zzxy	5.1	1.4	8.4	6.8
RMSD		–	1.1	2.4	1.4

[a] The "large" basis is def2-SVP for C, H and def2-TZVP for Mn, V, O. The "small" basis is def2-SVP for all atoms.

S8 Transition paths between Jahn–Teller Arrangements

The large number of JT arrangements for the minima also implies that there is a large number of transition states connecting those minima. In this section, we use symmetry to derive all distinguishable transition paths between the three stable minima of the Mn3344 oxidation state of the precatalyst.

As written in the main text, the Mn3344 oxidation state of the precatalyst has three distinct local minima (44xy, yz44, zz44, see Figure 4 and Table 2). Thus, one could think that there are only three transition states. However, in fact there are 12 minima when considering all degenerate minima (Figure S1, Table S1), too. In order to find all relevant transition paths, one needs to include all 12 minima. The minima are shown in Figure S6 (top). The figure is made in such a way that rotating or mirroring the figure has the same effect as rotating or mirroring the molecule (within the C_{3v} point group of the molecular framework). Thus, in the figure the three symmetry-equivalent minima 44xy, 4z4y, and 4zx4 are shown in equivalent positions and can be transformed into each other by rotating the figure by 120° or 240°. Likewise, the y44y, zz44, and x4x4 minima are related by rotations. The six other minima—x44y, z44y, yz44, xz44, z4x4, y4x4—can also be converted into each other by either mirroring or rotating.

As a next step, we identified all relevant transition pathways between these 12 minima. In principle, there are 66 ($\frac{1}{2} \cdot 12 \cdot 11$) possible pairs of minima, but some of these pairs refer to transformations where both JT axes change simultaneously. We assume that such transition pathways will be energetically unfavorable, compared to the pathways where only one JT axis needs to change. Hence, we identified the 39 pathways where this is the case; those are shown as grey lines in Figure S6.

The transition states themselves can also be interconverted into each other my the symmetry operations, and therefore it is possible to group the 39 pathways into classes of equivalent pathways. This leads to 8 classes of pathways, as shown in the bottom of Figure S6. We took advantage of this classification by selecting one pathway from each class (shown in thicker lines) and carried out transition state searches and potential energy scans only for those. The obtained eight reaction profiles are the ones shown in Figure 7 in the main text. Note that the number labels in the bottom of Figure S6 are the same as the ones used in Figure 7.

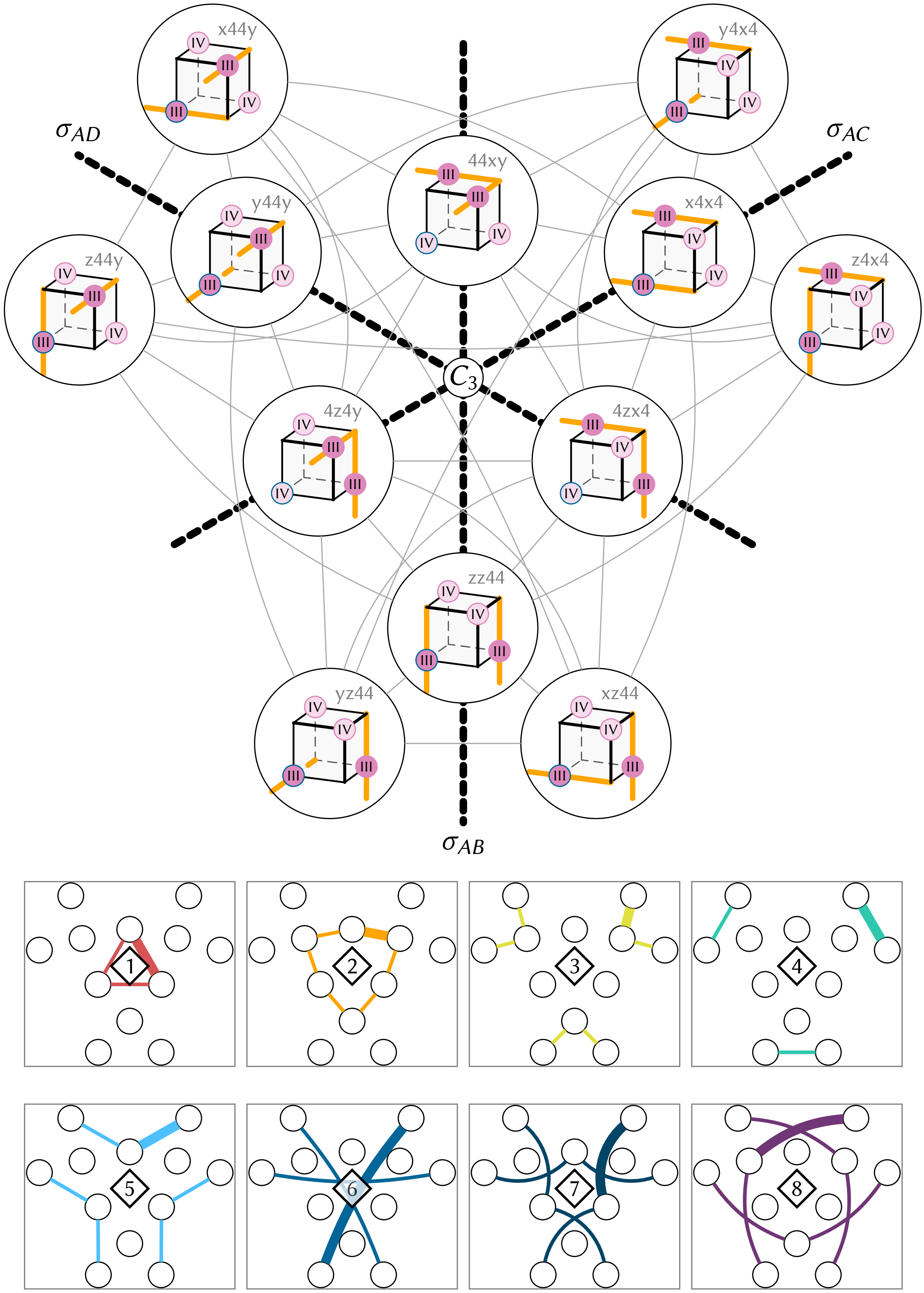

Figure S6: Symmetry relationships and transition paths between the all twelve stable JT arrangements of the precatalyst in the Mn3344 (S_1) oxidation state. Only transition paths are shown that involve the change of only one of the JT axes. The eight boxes show the eight distinguishable classes of transition paths; the number labels are the same as used in Figure 7. For explanations, see text.

S9 Absolute energies of reported geometries

Table S12: Absolute electronic, zero-point, and free energies of the stable minima of the precatalyst. The "index" refers to supporting xyz file "suppinfo_precatalyst.xyz".

Label	Conf.	Index	E_{elec} (E_h)	E_{ZP} (E_h)	G (E_h)
Mn4444	4444	1	-10348.047288	0.216756	-10347.830532
Mn3444	z444	2	-10348.212350	0.214330	-10347.998020
	4z44	3	-10348.228495	0.214955	-10348.013540
Mn3344	zz44	4	-10348.368004	0.212657	-10348.155347
	yz44	5	-10348.368483	0.212353	-10348.156130
	44xy	6	-10348.375339	0.212972	-10348.162367
Mn3334	z4xy	7	-10348.494473	0.210568	-10348.283905
	y4xy	8	-10348.494091	0.210266	-10348.283825
	4zxy	9	-10348.488804	0.210107	-10348.278697
	4yxy	10	-10348.491119	0.210774	-10348.280345
Mn3333	zzxy	11	-10348.588743	0.208087	-10348.380656
	zyxy	12	-10348.597529	0.208737	-10348.388792

Table S13: Absolute electronic, zero-point, and free energies of the stable minima of the catalyst. The "index" refers to supporting xyz file "suppinfo_catalyst.xyz".

Label	Conf.	Index	E_{elec} (E_h)	E_{ZP} (E_h)	G (E_h)
Mn4444	O4444	1	-10271.872569	0.203405	-10271.669164
	H4444	2	-10271.872036	0.203247	-10271.668789
Mn3444	O44x4	3	-10272.051781	0.201593	-10271.850188
	H44x4	4	-10272.050094	0.200494	-10271.849600
	O4z44	5	-10272.051331	0.201949	-10271.849382
	Oy444	6	-10272.039243	0.200958	-10271.838285
	Hy444	7	-10272.038698	0.200750	-10271.837948
	Hz444	8	-10272.039154	0.201254	-10271.837900
Mn3344	O4zx4	9	-10272.197443	0.199773	-10271.997670
	O44xy	10	-10272.196972	0.199655	-10271.997317
	Hx4x4	11	-10272.191947	0.197460	-10271.994487
	Oy4x4	12	-10272.192523	0.199043	-10271.993480
	Oxz44	13	-10272.192981	0.199575	-10271.993406
	Hy4x4	14	-10272.191401	0.198207	-10271.993194
	Hz4x4	15	-10272.190521	0.199570	-10271.990951
Mn3334	Oyzx4	16	-10272.317208	0.197227	-10272.119981
	Oy4xy	17	-10272.314648	0.197095	-10272.117553
	Hz4xy	18	-10272.313114	0.196681	-10272.116433
	O4zxy	19	-10272.309857	0.196572	-10272.113285
Mn3333	Oyzxy	20	-10272.417675	0.195676	-10272.221999

S10 Input parameters for Gaussian 16

The following input for Gaussian 16 has been used to obtain the absolute energies given in Tables S12 and S13. Some computations required additional settings for `opt` (some of `maxstep, gdiis, cartesian, calcfc`) or `scf` (some of `qc, xqc, damp, novaracc, noincfock, maxcyc`) in order to ensure proper convergence.

```
# p GFINPUT IOP(6/7=3)
# symmetry=none
# BP86/GEN
# opt=(tight) freq
# scf=(verytight)
# EmpiricalDispersion=GD3
# SCRF=(IEFPCM,solvent=acetonitrile)
# integral=(grid=superfinegrid)
# DenFit

Mn4O4 opt+freq

<charge> <multiplicity>
<geometry>

C H
def2SVP
****
Mn V O
def2tzvp
****
```
